# DeepRisk: A deep learning approach for genome-wide assessment of common disease risk

**DOI:** 10.1016/j.fmre.2024.02.015

**Published:** 2024-03-19

**Authors:** Jiajie Peng, Zhijie Bao, Jingyi Li, Ruijiang Han, Yuxian Wang, Lu Han, Jinghao Peng, Tao Wang, Jianye Hao, Zhongyu Wei, Xuequn Shang

**Affiliations:** a AI for Science Interdisciplinary Research Center, School of Computer Science, Northwestern Polytechnical University, Xi'an 710129, China; b Key Laboratory of Big Data Storage and Management, Northwestern Polytechnical University, Ministry of Industry and Information Technology, Xi'an 710129, China; c College of Intelligence and Computing, Tianjin University, Tianjin 300072, China; d School of Data Science, Fudan University, Shanghai 200433, China; e Research and Development Institute of Northwestern Polytechnical University in Shenzhen, Shenzhen 518000, China

**Keywords:** Disease risk prediction, Deep learning, Polygenic risk score, Common disease risk, Disease prevention

## Abstract

The potential for being able to identify individuals at high disease risk solely based on genotype data has garnered significant interest. Although widely applied, traditional polygenic risk scoring methods fall short, as they are built on additive models that fail to capture the intricate associations among single nucleotide polymorphisms (SNPs). This presents a limitation, as genetic diseases often arise from complex interactions between multiple SNPs. To address this challenge, we developed DeepRisk, a biological knowledge-driven deep learning method for modeling these complex, nonlinear associations among SNPs, to provide a more effective method for scoring the risk of common diseases with genome-wide genotype data. Evaluations demonstrated that DeepRisk outperforms existing PRS-based methods in identifying individuals at high risk for four common diseases: Alzheimer's disease, inflammatory bowel disease, type 2 diabetes, and breast cancer.

## Graphical abstract

**Figure:**
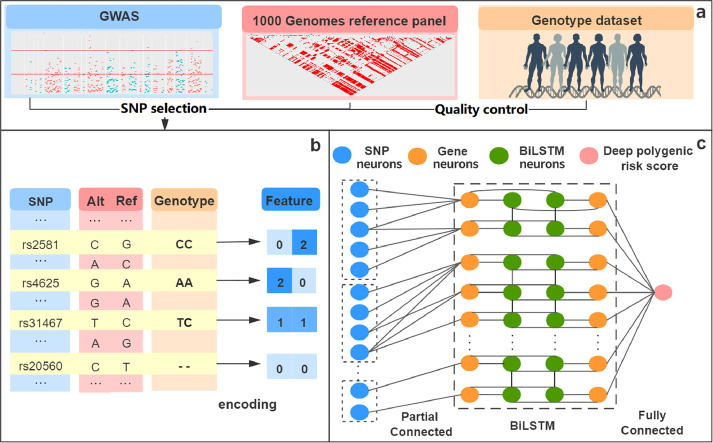

## 1 Introduction

A major public health need is the identification of high-risk individuals for a given disease, which can enable better screening or therapies [1]. For most human diseases, individual susceptibility is influenced by genetic variation to some extent [2]. Consequently, one important approach for identifying individuals at high risk is to stratify individuals based on inherited DNA variation [1]. According to the number of genes that cause the disease, diseases involving genetic factors are traditionally divided into single-gene Mendelian diseases and complex or common diseases [3]. In the 1980s and 1990s, based on linkage analysis and fine mapping within large multiplex pedigrees, efforts to map disease genes focused mainly on rare diseases, monogenic diseases and syndrome-type diseases [1]. Approximately 1,000 single-gene inherited diseases had been characterized, including many diseases that have a significant impact on biomedicine, such as Huntington's disease [4,5] and cystic fibrosis [6,7]. However, linkage analysis is very limited for common, later-onset traits associated with complex diseases, such as asthma, diabetes and depression [8]. Until 2005, genome-wide association studies (GWASs) identified a large number of genetic variants, mostly single nucleotide polymorphisms (SNPs) [9]. The emergence of GWASs provided important clues for the discovery of genetic characteristics that influence the occurrence of complex diseases [3].

In the decades since the first GWAS [10], people's understanding of the genetic basis of common human diseases has changed. For complex or common diseases, genetic susceptibility is jointly determined by thousands of common variants, and a single variant has little effect on population risk [11,12]. On the basis of GWAS, the polygenic risk score (PRS), which quantifies individual genetic risk, has become a powerful tool for common disease risk prediction [13]. The PRS quantifies the degree of individual susceptibility to disease by calculating the cumulative effect of multiple susceptibility sites [14]. The development of robust PRSs for several common diseases has been catalyzed by the continuous expansion of the GWAS dataset scale and the establishment of large-scale biobank support score verification [1,^2^,^15^,16]. Many studies have demonstrated the utility of PRSs for disease risk stratification as well as their implications for early disease detection, prevention, therapeutic intervention and life planning [17]. The traditional PRS-based method quantifies the impact of variations on individuals based on a simple linear additive model. However, the mechanisms of gene action and the structure of complex traits are actually much more complex than described by the additive model [18]. For example, epistasis, functionally defined as an event where the influence of one locus depends on the genotype of another, is a type of nonadditive association [19]. A study showed that there is an interaction effect between HLA-C and ERAP1 in psoriasis, and ERAP1 variants influence psoriasis susceptibility only in individuals carrying the HLA-C risk allele [20]. Therefore, the traditional additive method of PRS calculation for risk identification not only fails to utilize the location information of variations but also ignores the nonlinear interaction information among various variations. In addition, traditional methods are constructed using classifiers such as logistic regression model [21,22] and penalty regression model [23]. These models struggle to adequately represent the nonlinear associations among SNPs, limiting their fitting capabilities and thereby affecting the accuracy of the final prediction results.

Recently, deep learning, a subfield of machine learning, has been successfully applied in several areas, such as medical imaging, health record processing and generalized deep learning methods for genomics [24], [25], [26], [27].

Inspired by the success of deep learning in the health care industry, we hypothesize that deep learning can further enhance the predictive ability of risk identification models by integrating large-scale genotype data. Research has shown that disease risk may be related to nongenetic factors [28]. For example, age is a recognized important factor that increases the risk of Alzheimer's disease. In this study, we present DeepRisk, an efficient method for disease risk prediction inspired by biological knowledge. This approach allows for the calculation of an individual's genetic risk and stratification of the population. DeepRisk incorporates a bidirectional long short-term memory network (BiLSTM) as a classification model and combines genotype features with additional data to improve the accuracy of disease risk prediction. Going beyond current methods, DeepRisk considers not only the positional relationships of SNPs but also the knowledge of SNP-gene associations to construct a part of the network that informs the predictive model while harnessing the power of BiLSTM to capture interaction information between long-distance genes on each chromosome, consequently enhancing its ability to capture complex genetic interactions. The risk analysis demonstrated that the disease risk of people with high risk scores according to our method was much greater than that of people with low risk scores. These findings indicate that DeepRisk can be used as an early warning tool for disease prevention and screening in high-risk populations.

## 2 Materials and methods

### 2.1 Overview of the DeepRisk model

**Fig. 1:**
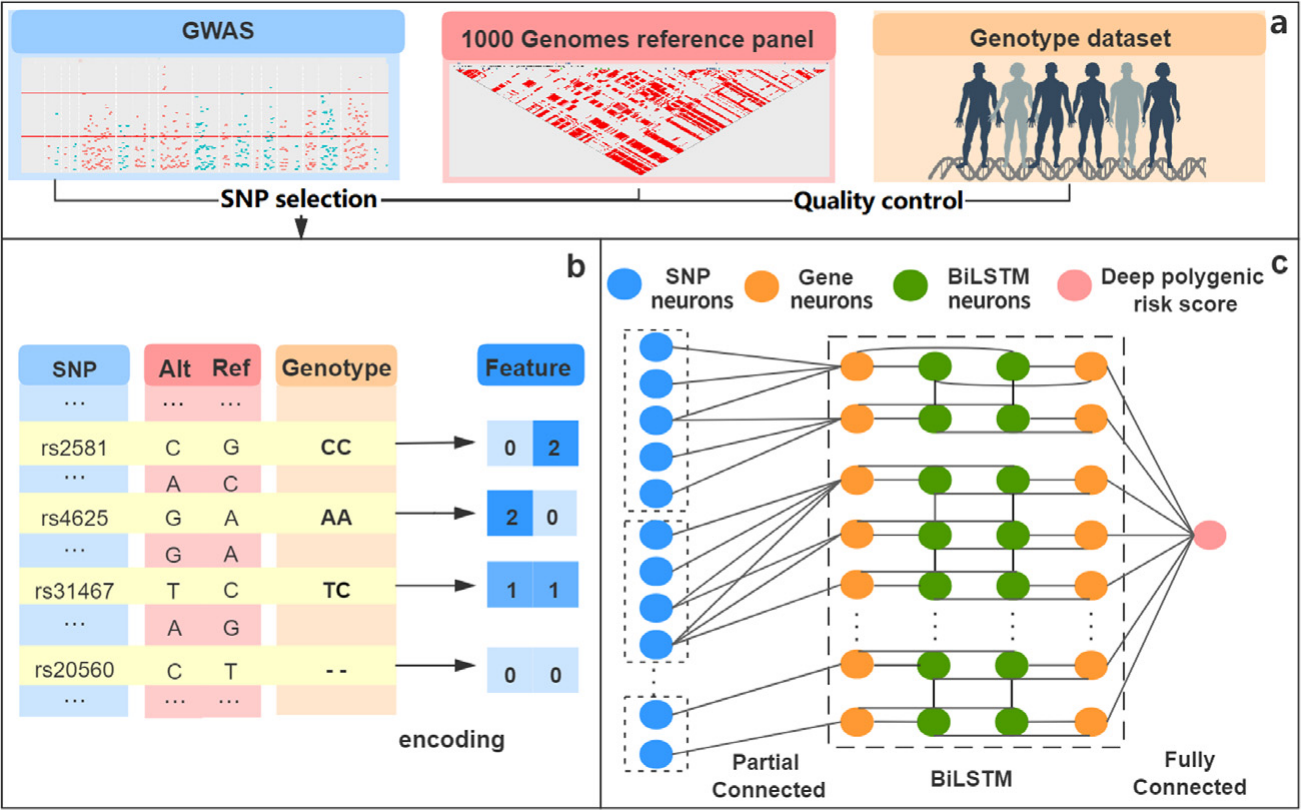
**Overview of the DeepRisk method**. DeepRisk includes three main components. (a) SNP selection step considers the significance SNPs based on the GWAS result, the linkage disequilibrium reference panel from 1000 Genomes Europeans and the availability of genotype data. (b) The genotype information is encoded into two dimensions of feature vectors, where the first dimension represents the number of non-risk allele (Ref allele), the second dimension represents the number of risk allele (Alt allele), the value of both dimensions of missing genotype is set to zero. (c) We first group SNP neurons according to chromosomes, then adopt a partial connected layer to connect SNP feature vectors to corresponding genes and use BiLSTM to capture the interaction relationship between genes. The deep polygenic risk score is obtained based on the fully connected layer with a sigmoid function.

In addition, DeepRisk combines genotype data [29], summary statistics from GWASs [30], [31], [32], [33], and data from the 1,000 Genomes reference panel [34] to predict the risk of common diseases (Figs. 1a, S6). In brief, we first carried out quality control (QC) on the genotype dataset, and then, a set of parameters based on GWAS results (*p* values) and the linkage disequilibrium reference panel from the 1000 Genomes of 503 Europeans (*r^2^*) were applied to select SNPs. The feature selection of this step provides high-quality and low-dimensional input for generating the risk score, which can greatly reduce the number of parameters and running time of the neural networks. Then, an encoding schema is proposed to represent genotype information. The two dimensions of the feature vector represent risk alleles (Alternative allele) and non-risk alleles (Reference allele). Compared with the traditional additive encoding method, our method not only represents the number of alleles carried but also avoids superfluous quantitative relationships, allowing us to more effectively handle missing genotypes (Fig. 1b). Finally, the features are fed to deep neural networks for disease risk prediction. Capitalizing on biological knowledge of SNP–gene associations, we first constructed a network layer by connecting SNPs to corresponding genes using a partially connected layer. This approach not only greatly reduces the feature dimension and effectively mitigates the phenomenon of overfitting but also infuses our model with prior biological knowledge, which further enhances our model's capacity to capture the intricate associations among SNPs and between SNPs and diseases. Next, we use the BiLSTM layer to capture the interaction information between long-distance genes from the forward and backward directions at the same time. Finally, we use the fully connected layer as the classifier. The prediction probability was used as the deep learning-based polygenic risk score (Fig. 1c).

### 2.2 Data preprocessing

#### 2.2.1 Quality control of the UK Biobank genotype dataset

The UKB project is a large prospective cohort study of ∼500,000 individuals across the United Kingdom aged between 40 and 69 at recruitment [35]. The genotypes of 488,377 UKB participants were obtained using two very similar genotyping arrays (the UK BiLEVE Axiom Array and the UK Biobank Axiom Array), which consist of more than 800,000 genetic markers. The quality control (QC) process included SNP-based QC and sample-based QC [1,36] (Supplementary Fig. S5). To identify poor-quality markers, we used the SNP quality control information released by the UKB (Resource 1955) [29] and selected SNPs using the following criteria. SNPs were measured on both genotyping arrays; SNPs that passed QC in more than 95% of the batches based on Resource 1955; and SNPs in 22 autosomal regions. In addition, DNA polymorphisms with ambiguous strands (A/T or C/G) were removed. We applied sample-based QC to identify individuals of British ancestry based on self-reported ancestry and genetically confirmed ancestry using a principal component analysis (PCA)-based method [36]. In addition, we applied sample-based QC to identify poor-quality samples for which the heterozygosity or genotype missing data were not reported, discordant reported versus genotypic sex, chromosomal anomalies, or ten or more third-degree relative identified individuals (kinship coefficient = 10) or for whom informed consent was withdrawn [1,29]. All the samples that failed QC were removed from the subsequent analysis. As a result, 408,308 samples and 665,207 SNPs passed the QC process.

#### 2.2.2 Determination of the case group and control group

The diagnosis of prevalent disease was based on a composite of self-reported data from an interview with a trained nurse, electronic health record information, including inpatient International Classification of Diseases (ICD-9 and ICD-10) diagnosis codes, and first occurrence of health outcome codes [1,[36], [37], [38], [39], [40]. To avoid the occurrence of false-negatives, healthy people in the control group were not only rescreened based on the above criteria but also excluded from the sample whose parents had disease based on family history records to avoid the influence of genetic factors (Figs. S1-S4).

The diagnosis of Alzheimer's disease was based on International Classification of Disease 10 (ICD-10) codes (F00 or G30) in hospitalization records or the first reported codes (130836, 131036, or 42020) [36]. The control group excluded individuals with unspecified dementia, mild cognitive impairment, other unclassified neurodegenerative diseases, and parents with Alzheimer's disease; the inclusion of the ICD-9 code (331, 290, or 2941) or the ICD-10 code (G31, F03, or F067) in hospitalization records; self-reports from an interview with a trained nurse (20002.1263); the date of first reported disease (131038, 130842, or 42018); or parents with Alzheimer's disease (20107.10 or 20110.10) (Fig. S1) [36].

Inflammatory bowel disease was ascertained based on International Classification of Disease-9 (ICD-9) codes (555 or 556) and International Classification of Disease-10 (ICD-10) codes (K50 or K51) in hospitalization records; self-reports in an interview with a trained nurse (20002.1461–1463); or the first reported codes (131626 or 131628) [1], [38]. The control group excluded individuals with other noninfective gastroenteritis and colitis conditions, including those with an ICD-9 code (558) or an ICD-10 code (K52) in hospitalization records or self-reports from an interview with a trained nurse (20002.1459) (Fig. S2) [1], [38].

The type 2 diabetes diagnosis was based on an ICD-10 code (E11) in the hospitalization records, self-reports in an interview with a trained nurse (20002.1223) or the first reported code (130708) [1], [37], [39]. The control group excluded individuals with other specified diabetes mellitus conditions, including an ICD-9 code (250) or an ICD-10 code (E10, E12-14, O24, E232, N083, N251) in hospitalization records, or self-reports from interviews with trained nurses (20002. 1220–1222, or 20002.1521), the first reported codes (130706, 130710, 130712, 130714, 132202, 2976, 2443, 4041 or 10844) or a family history of diabetes (20107.9 or 20110.9) (Fig. S3) [1], [37], [39].

Breast cancer diagnosis was based on self-reports from interviews with a trained nurse (20001.1002), International Classification of Disease-9 (ICD-9) codes (174) or International Classification of Diseases 10th Edition (ICD-10) codes (C50) in hospitalization records [1,40]. The control group excluded samples with other neoplasms or carcinomas in situ of the breast, including those with an ICD-9 code (217 or 2330) or an ICD-10 code (C or D05) in hospitalization records, or self-reports from interviews with trained nurses (20001. X), the first reported code (40005), or a family history of breast cancer (20110.5) (Fig. S4) [1], [40].

#### 2.2.3 Additional features

Inspired by established research, we selected additional features for our study, including population characteristics such as age, sex, region of the assessment center, Townsend Deprivation Index, educational qualifications, and the first four genetic principal components, as well as technical covariates like genotype measurement batch and genotype array [1,^36^,^38^,41].

### 2.3 The DeepRisk method

The method contains three main components: SNP selection (Fig. 1a), SNP feature encoding (Fig. 1b) and a disease risk prediction module (Fig. 1c).

#### 2.3.1 SNP feature selection module

First, we performed SNP selection on the UKB dataset after preprocessing based on summary statistics from recent GWASs conducted primarily among participants of European ancestry for four diseases [30], [31], [32], [33] and a linkage disequilibrium reference panel of 503 European samples from the 1000 Genomes phase 3 version 5 [34]. UKB samples were not used in any of the four GWASs. The *p* values were obtained from recent GWAS summary statistics. *r^2^* was built using a linkage disequilibrium-driven clumping procedure with a moving window of size 250 kilobases based on PLINK version 1.90b [42]. The final output of the clumping procedure included the most significant disease-associated SNP for each linkage disequilibrium-based clump across the genome. We set up a range of *p* values (5 × 10^−3^, 5 × 10^−4^, 5 × 10^−5^, 5 × 10^−6^) and *r^2^* thresholds (0.2, 0.4, 0.6, 0.8, -) for SNP selection. The “-” symbol specifically indicates instances where the *r^2^* threshold is not applied in the selection process of SNPs. These parameters are used to generate 20 candidate DeepRisk scores for the training set. We choose a pair of parameters that perform best on the training set and apply them to the testing set.

#### 2.3.2 SNP feature encoding module

Based on the genotype dataset obtained in the previous step, the SNP genotype can be represented by the number of alternative alleles and reference alleles. The traditional one-dimension method only encodes the number of alternative alleles. For example, 0, 1 and 2 represent none, one and two alternative alleles respectively. This kind of cumulative numerical coding might introduce a quantitatively biased assumption like there is a twofold relationship between a homozygous genotype and a heterozygous genotype, which could negatively influence the modeling of non-linear relationships. The four-dimension way, known as one-hot encoding, represents the features (including 0, 1, 2, and missing) sparsely as a four-dimension binary vector [43]. Although this method avoids quantitative bias, it increases the number of parameters in the model. To address these issues, our coding strategy uses two dimensions for genotype encoding. One dimension represents alternative allele and the other one represents reference allele. In particular, both dimensions are set to zero for missing genotypes. Compared to one or four-dimension way, the proposed method can not only maintain the independence between features of reference allele and alternative allele but also keep a simplified model structure.

#### 2.3.3 Disease risk prediction module

Based on the SNP genotype features obtained in the previous step, these features are divided into 22 groups according to chromosome. We input the features into the neural network according to the chromosomes. The dimensions of the original SNP features are relatively large, which may cause severe overfitting. To overcome this problem, we implement a partial connection layer, which serves as a crucial component in our model. This layer is designed to establish connections between each SNP and nearby genes located within a 250 kb range both upstream and downstream. Each node in this layer represents a gene. If a SNP does not map to any gene, we link the SNP to its nearest gene, ensuring that every SNP connects to at least one gene. It is noted that a gene can connect multiple SNPs. Similarly, an individual SNP can connect to multiple genes, recognizing the polygenic influence of SNP. Given m SNPs in a gene region, the feature of this gene can be calculated by aggregating the features of these SNPs as follows:

(1) $$F_{gene}=\left(W^{T}F_{SNP}\right)$$

where $F_{\mathrm{SNP}}\in R^{2m\;\times \;1}$ represents the SNP feature, $F_{gene}\in R^{1\;\times \;1}$ represents the gene feature, and $W\in R^{2m\times \;1}$ is a trainable weight matrix for SNP features toward the corresponding gene.

After the partially connected layer, the SNP features are transformed into gene features, which are subsequently fed to the BiLSTM layer to capture the distance interaction. BiLSTM consists of two LSTM components, which process the input in the forward and backward directions [44]. Given the n genes on a chromosome and the features of these genes $F_{\mathrm{gene}}\in R^{n\;\times \;1}$, we input each gene feature at each step. For example, at step t, given the input gene feature vector $x_{t}$, the hidden state $h_{t}$ can be obtained as follows:

(2) $${\vec{h}}_{t}=\vec{LSTM}\left(x_{t},{\vec{h}}_{t-1}\right)$$

(3) $${\overset{\leftarrow }{h}}_{t}=\overset{\leftarrow }{LSTM}\left(x_{t},{\overset{\leftarrow }{h}}_{t-1}\right)$$

(4) ht=(h→t,←ht)

where ${\vec{h}}_{t}$ is the hidden state of the forward LSTM and ←ht is the hidden state of the backward LSTM. $h_{t}$ is a more information-enriched vector obtained by extracting forward and backward interaction information from all the genes on a chromosome. The final output H *is obtained by* combining steps h:

(5) $$H=\left(h_{0},h_{1}\cdots h_{t}\cdots h_{n-1}\right)$$

We set the hidden units of each unidirectional LSTM as four. To avoid overfitting, we add L2 regularization [45] and dropout [46] to the BiLSTM layer. Finally, the deep polygenic risk score was obtained based on the fully connected layer:

(6) $$score=\sigma \left(W^{T}x_{i}+b\right)$$

where $\sigma \left(x\right)=1/\left(1+e^{-x}\right)$ is the sigmoid function [47], $x_{i}$ is the output of BiLSTM, and W and b are trainable parameters. Since this is a classification problem with extremely unbalanced classes, we use the binary cross-entropy loss with class weights to train the model [48]. The loss function is as follows:

(7) $$L_{weighted}\;=\;-\omega_{1}y\log\left(p\right)-\omega_{0}\left(1-y\right)\log\left(1-p\right)$$

where $\omega_{0}\;=\;N_{samples}/\left(2*(N_{samples}-N_{positive})\right)$ is the weight for the negative class, $\omega_{1}\;=\;N_{samples}/\left(2*N_{positive}\right)$ is the weight for the positive class, $N_{samples}$ is the number of samples, $N_{positive}$ is the number of samples in the positive class, y is the binary label (0 or 1) and p is the predicted probability.

Finally, we added additional features as the input to test the effect of additional features for disease risk prediction. The deep polygenic risk score with additional features was determined using the DeepRisk model with genotype, age, sex, genotype measurement batch, genotype array, region of assessment center, Townsend Deprivation index at recruitment, education-qualifications, and the first four genetic principal components. The model architecture of DeepRisk with additional features can be found in the Supplementary Information (Fig. S6).

### 2.4 Odds ratio calculation

To assess the relative risk of individuals with a high deep polygenic risk score and those with a low deep polygenic risk score, we used the odds ratio index for risk analysis. After obtaining the deep polygenic risk score via DeepRisk, we ranked the scores in descending order. For illustration, we describe the method using 20% and 80% as cut-offs in the following description. Specifically, T represents the total number of individuals in the top 20% of the distribution; $T_{D}$ individuals developed the disease, and $T_{E}$ individuals remained healthy. R represents the total number of remaining 80% individuals, in which $R_{D}$ individuals developed the disease and $R_{E}$ individuals remained healthy (Table S1). The odds ratio was calculated as follows:

(8) $$odds\;ratio=\frac{T_{D}}{T_{E}}/\frac{R_{D}}{R_{E}}=\frac{T_{D}R_{E}}{T_{E}R_{D}}$$

If OR>1, the deep polygenic risk score is positively correlated with the risk of disease. In addition, individuals were binned into 100 groups according to the percentile of the deep polygenic risk score, and the prevalence of disease within each bin was determined by the number of patients in the group versus the total number of people in the group. In statistics, a percentile is a rank score below which a given percentage of scores in its frequency distribution falls [49].

### 2.5 Evaluation metrics

We adapted the area under the receiver operating characteristic curve (AUC) as a performance metric for model performance evaluation [50]. Based on the tenfold cross-validation method [51] used to divide the dataset, we compared DeepRisk with the pruning and thresholding method [21] and lasso model [23]. The pruning and thresholding methods and lasso method can be found in the Supplementary Document.

## 3 Results

### 3.1 Overall performance of DeepRisk

We tested DeepRisk on four common diseases, namely, Alzheimer's disease (AD), inflammatory bowel disease (IBD), type 2 diabetes (T2D) and breast cancer (BC). The basic information of the dataset is shown in Table 1. The area under the curve (AUC) of the deep polygenic risk score was 0.7245, 0.6517, 0.6508 and 0.6227 for Alzheimer's disease, inflammatory bowel disease, type 2 diabetes and breast cancer, respectively (Table 1). The deep polygenic risk score with additional features also performed well in the test dataset, with AUCs of 0.8624, 0.6585, 0.7316 and 0.6660 for Alzheimer's disease, inflammatory bowel disease, type 2 diabetes and breast cancer, respectively (Table 1).

**Table 1:** **Deep polygenic risk score derivation and testing for four common diseases**. Additional features include age, sex, genotype measurement batch, genotype array, region of assessment center, Townsend Deprivation index at recruitment, education-qualifications, the first four of genetic principal components. The breast cancer analysis is restricted to female participants.

Disease	Discovery GWAS (n)	Number of SNPs	DeepRisk AUC	DeepRisk AUC with additional features
AD [30]	17,008 cases 37,154 controls	771	0.7245	0.8624
IBD [31]	12,882 cases 21,770 controls	2,481	0.6517	0.6585
T2D [32]	26,676 cases 132,532 controls	5,968	0.6508	0.7316
BC [33]	122,977 cases 105,974 controls	3,830	0.6227	0.6660

### 3.2 DeepRisk can identify individuals at high risk for AD, IBD, T2D and BC

**Fig. 2:**
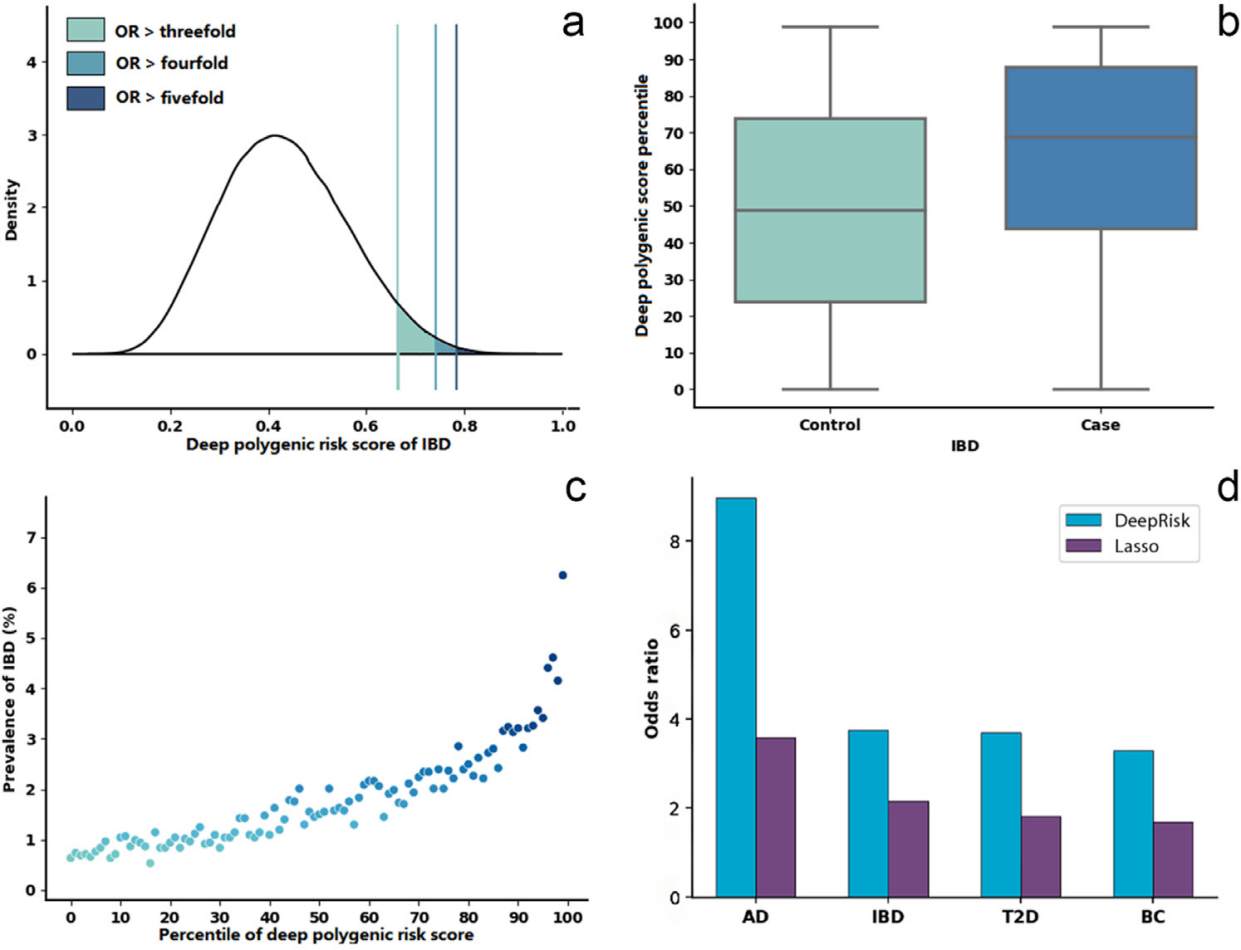
**Risk analysis for IBD based on DeepRisk**. (a) Distribution of deep polygenic risk score of IBD in the UK Biobank dataset. The *X*-axis represents deep polygenic risk score of IBD. Shading reflects the proportion of the population with three-, four-, and fivefold increased risk versus the remainder of the population. (b) Deep polygenic risk score percentile among IBD cases versus controls in the UK Biobank dataset. In each boxplot, the horizontal lines reflect the median, the top and bottom of each box reflect the quartile range, the whiskers reflect the maximum and minimum values within each group. (c) Prevalence of IBD according to 100 groups of the dataset binned according to the percentile of the deep polygenic risk score of IBD. (d) Odds ratio performance of DeepRisk and Lasso-based method on four diseases, sort individuals by polygenic risk score and use the top 1% of them versus the others.

We performed a risk analysis to test whether DeepRisk can identify individuals with high disease risk. Like in the previous study [1], a given threshold was used to group the individuals based on the deep polygenic risk score. The odds ratio (OR) was calculated to compare the risk level between individuals with high risk scores and individuals with other risk scores (see details in the Materials and methods section). Taking IBD as an example, we found that 4.19% of the population inherited a genetic predisposition, with a more than threefold increased risk for IBD. Furthermore, 0.9% of the population has a more than fourfold increased risk for IBD, and 0.29% has a more than fivefold increased risk (Fig. 2a; Table 2). The median DeepRisk_IBD_ percentile score was 69 for individuals with IBD, which was much greater than the 49 for non-IBD individuals (Fig. 2b). We separated the individuals into 100 groups according to the percentile of the deep polygenic risk score. The risk of IBD increases sharply in the right tail of the deep polygenic score distribution, from 0.64% in the lowest percentile to 6.24% in the highest percentile, which indicates that DeepRisk can identify individuals at high risk (Fig. 2c). Similar results were also found for AD, BC and T2D (Figs. S8-10). The proportions of the population with ORs greater than five were 7.90%, 0.06% and 0.15% for AD, BC and T2D, respectively (Figs. S8a-10a). The median DeepRisk percentile scores were 81, 65, and 67 for individuals with AD, BC and T2D, respectively, which were much greater than the 49, 47, and 48 for non-AD, non-BC and non-T2D individuals, respectively (Figs. S8b-10b). The risks of AD, BC and T2D all increased sharply in the right tail of the deep polygenic score distribution, from 0.17%, 2.85%, and 2.31% in the lowest percentile to 4.70%, 23.13%, and 26.10% in the highest percentile, respectively (Figs. S8c-10c). Furthermore, DeepRisk performed better than the lasso model for different given thresholds for defining high-risk individuals (Table S2).

**Table 2:** **Proportion of the population at three-, four- and fivefold increased risk for Alzheimer's disease, inflammatory bowel disease, type 2 diabetes and breast cancer**.

High PRS definition	Individuals in UKB dataset (n)	% of individuals
**Odds ratio ≥ 3.0**		
Alzheimer's disease	243,567/351,022	69.39
Inflammatory bowel disease	16,457/392,613	4.19
Type 2 diabetes	14,190/346,333	4.10
Breast cancer	2,731/172,160	1.59
**Odds ratio ≥ 4.0**		
Alzheimer's disease	140,151/351,022	39.93
Inflammatory bowel disease	3,515/392,613	0.90
Type 2 diabetes	2,283/346,333	0.66
Breast cancer	328/172,160	0.19
**Odds ratio ≥ 5.0**		
Alzheimer's disease	27,706/351,022	7.90
Inflammatory bowel disease	1,138/392,613	0.29
Type 2 diabetes	532/346,333	0.15
Breast cancer	103/172,160	0.06

### 3.3 DeepRisk performs better than the other two existing methods on four diseases

We compare DeepRisk with two existing methods, the pruning and thresholding method and the lasso-based method, for four diseases, namely, AD, IBD, T2D and BC. The pruning and thresholding methods and lasso-based methods used can be found in the Supplementary Materials.

Compared with the other two state-of-the-art methods, the results show that DeepRisk achieves the best performance when using only the genotype or when the genotype is combined with additional features as input for all four diseases. First, following the metric in the previous subsection, DeepRisk performed better than the lasso model for different given thresholds for defining high-risk individuals (Table S2). For the top 1% of the population versus the others, using the deep polygenic risk score, the ORs of AD, IBD, T2D and BC were 8.97, 3.75, 3.70 and 3.29, respectively. According to the lasso model, the ORs were much lower, at 3.58, 2.15, 1.81 and 1.68 for AD, IBD, T2D and BC, respectively (Fig. 2d). DeepRisk performs significantly better than does the lasso-based method for all diseases. Then, we compare DeepRisk with the existing methods based on the area under the receiver operating characteristic curve (AUC), which is a metric usually used for machine learning tasks [50]. The patients in the UKB dataset were divided into a case group and a control group according to the specific disease (Table 1 and Figs. S1-4). To avoid circular logic, we used GWAS outputs that did not include the UKB dataset. Taking AD as an example, SNP selection was based on a recent GWAS involving 54,162 participants and a linkage disequilibrium reference panel of 503 Europeans from 1000 Genomes (Table 1). After quality control, the UK Biobank dataset included 351,022 participants, 2,066 of whom were diagnosed with AD (Table 1 and Fig. S1). When *p* < 0.0005 and *r^2^* < 1, the prediction performance was the best, with 771 variants (Table 1). For disease risk prediction, we used tenfold cross-validation [51] for performance comparison. We predicted disease risk based on genotype only and on genotypes with additional features using three methods, namely, the DeepRisk, pruning and thresholding methods [21], and the lasso model [23] (Tables S3-10). The results showed that DeepRisk consistently performed better than the other two methods (Fig. S7). Furthermore, the results indicate that our method is not sensitive to linkage disequilibrium parameters (Tables S2-9), indicating that DeepRisk can automatically address the redundant information between different SNPs without prior information for linkage disequilibrium filtering.

### 3.4 DeepRisk can identify high-risk individuals with a small number of risk SNPs

**Fig. 3:**
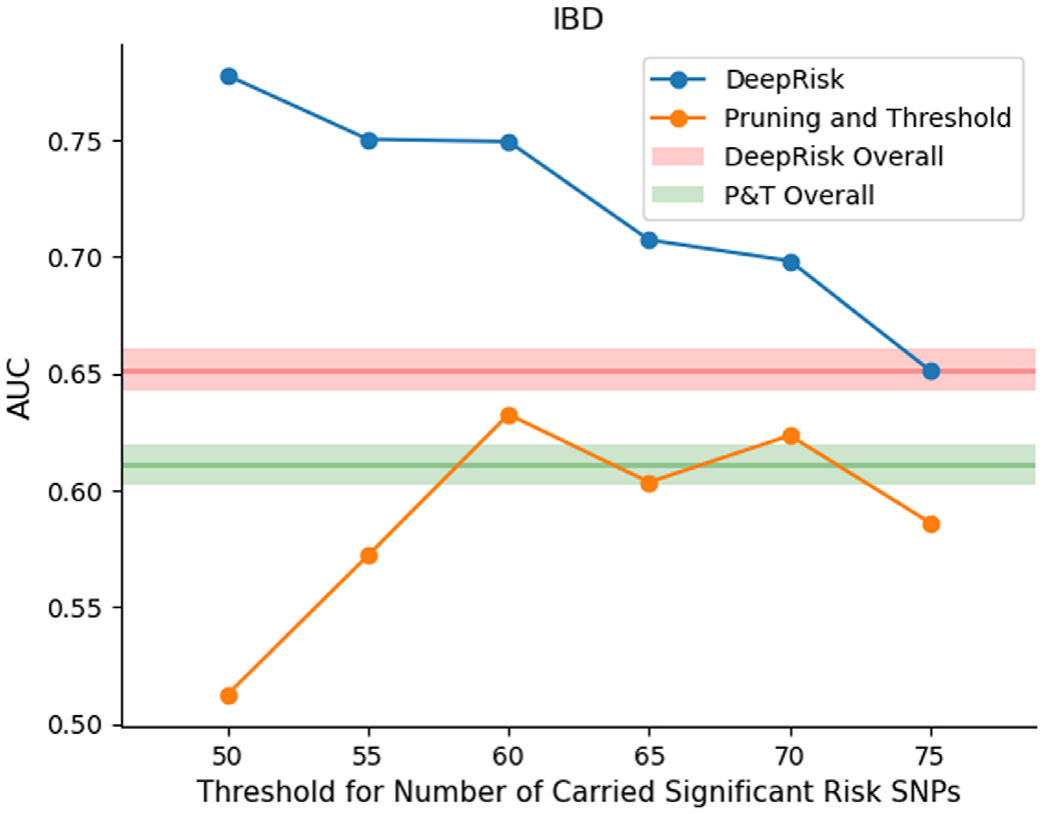
**Comparison of DeepRisk and P&T method performance in subpopulations with a small number of risk SNPs**. The figure shows the AUC performance of each method in the 1% individuals with fewest number of risk SNPs of Inflammatory Bowel Disease (IBD). The cut-off corresponding to the 1% individuals with fewest number of risk SNPs is 75 risk SNPs. The *X-*axis represents the cut-off number of carried risk SNPs, and the *Y-*axis represents the AUC score.

The traditional weighted-sum methods for calculating PRS are mainly based on the number of risk SNPs and their effect estimate (*β*) from GWAS results. To assess the ability of DeepRisk in predicting disease risk for individuals with a small number of risk SNPs, we further analyzed the prediction result of IBD made by DeepRisk and pruning and threshold method (*P* + *T*). We used individuals and the corresponding prediction result of the ten-fold cross-validation from Section 3.3. We only used the prediction results based on the genotype data. The area under the receiver operating characteristic curve (AUC) is utilized to evaluate the performance of algorithms on individuals with only a small number of risk SNPs. We considered SNPs with GWAS *p* values below 5e-8 and a positive effect size as risk SNPs. An individual is considered to carry a risk SNP if they possess at least one alternative allele for this risk SNP. We ranked individuals based on the number of risk SNPs they carried and focus on the top 1% of individuals with the least number of risk SNPs. This corresponds to a cut-off of 75 risk SNPs. Additionally, we also set different cut-offs for individuals with fewer than 75 risk SNPs to show corresponding performance. The individuals were grouped as subgroups by various cut-offs. The results show that DeepRisk performs better than the pruning and thresholding method on the individuals with a small number of risk SNPs (Fig. 3), indicating the superior performance of DeepRisk. For example, in the subgroup with not greater than 75 risk SNPs, the AUC for DeepRisk was 0.651 compared to 0.586 for *P* + *T*, indicating a 1.6 times larger difference compared to the difference observed on the whole dataset. Similar results are observed on the subgroups with fewer risk SNPs. We also found some cases in our study, which can support the systematic analysis (Table S11). For instance, some individuals with IBD who carry few risk SNPs had low PRS percentile scores according to the pruning and thresholding methods. In contrast, their DeepRisk percentile scores were much higher. This shows the potential of DeepRisk to identify high-risk individuals with few risk SNPs.

### 3.5 Each component of DeepRisk is well designed for disease risk prediction

To illustrate the contribution of each part of DeepRisk to the prediction performance, we tested three methods by replacing the other three parts of DeepRisk, namely, using the traditional additive encoding method to replace our encoding schema (method a), directly using SNP features for subsequent feature extraction without the partially connected layer (method b), and using a CNN to replace BiLSTM to extract gene features (method c). In this test, we use only genotype features as inputs and compare different methods based on tenfold cross-validation. The results showed that the performance of DeepRisk was better than that of the other three methods, indicating that the model was designed properly (Table 3). Compared with the additive encoding schema of method a, the reason for the superior performance of DeepRisk may be that the new encoding method expands the dimension of the feature. The traditional encoding method uses 0, 1 and 2 to represent genotypes, which is likely to bring additional quantitative relationships. In fact, there may not be a twofold risk relationship between a homozygous genotype and a heterozygous genotype. We removed this additional quantitative relationship and better handled the missing genotype by filling it with (zero, zero) instead of deleting the locus. Compared with method b, DeepRisk uses a partially connected layer to connect the SNP features to the neurons representing their adjacent genes. This approach not only greatly reduces the number of parameters and running time of deep neural network models but also takes the association between SNPs and genes into account. In addition, the effect of SNPs on individuals is ultimately expressed by genes, and it is of greater biological significance to extract features using a gene-based partially connected layer. Compared with the CNN model of method c, the superior performance of DeepRisk may be because the CNN can extract information only about local SNP interactions, while BiLSTM can capture SNP interaction information at a distance on a chromosome.

**Table 3:** **The effect of each component of DeepRisk on disease risk prediction**. Evaluation with AUC metrics.

Disease	DeepRisk AUC	Method a AUC	Method b AUC	Method c AUC
Alzheimer's disease	0.7245	0.7158	0.7216	0.7232
Inflammatory bowel disease	0.6517	0.6485	0.6469	0.6473
Type 2 diabetes	0.6508	0.6490	0.6440	0.6457
Breast cancer	0.6227	0.6169	0.6232	0.6162

### 3.6 DeepRisk is robust to the variation in significant SNPs for disease risk prediction

**Fig. 4:**
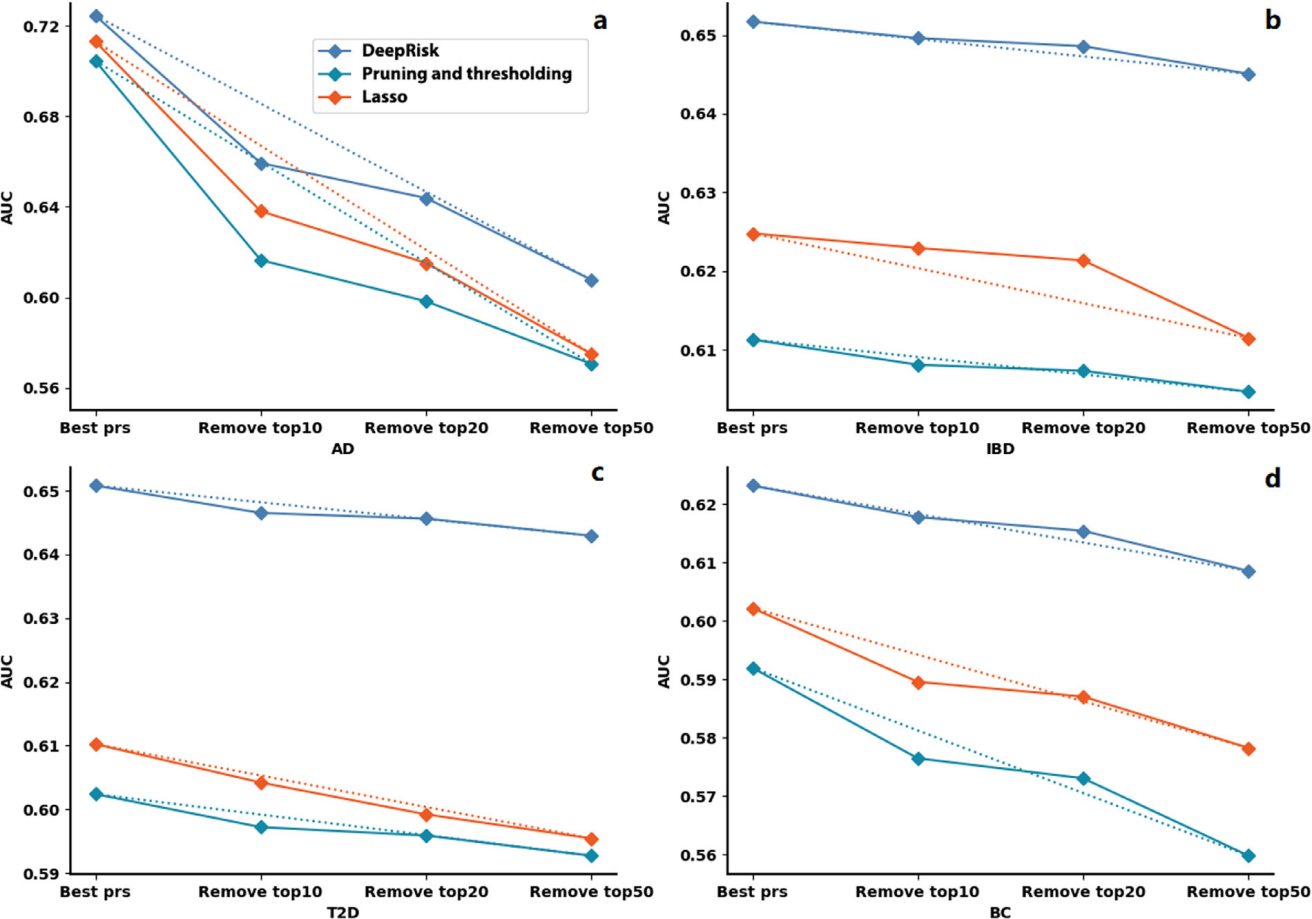
**The results of risk prediction for Alzheimer's disease (a) inflammatory bowel disease (b) type 2 diabetes (c) and breast cancer (d) after removing different significant SNPs**. The dotted line indicates the rate of performance degradation. The *X*-axis represents the number of significant SNPs removed in different evaluation tests, and the *Y-*axis represents the AUC score.

At present, GWAS-based methods cannot identify all significant disease-related loci. However, the DeepRisk, pruning and thresholding and the Lasso method require the selection of significant SNPs based on the *p* values given by the GWAS. Therefore, we tested whether the absence of significant SNPs affects the performance of these three methods. We sequentially removed the most significant SNPs in the top 10, top 20 and top 50 from the GWAS results. The results showed that DeepRisk performed consistently better than the other two methods (Fig. 4). Although the AUCs of all three methods decrease with the increase in missing significant SNPs, DeepRisk is the most robust method (Fig. 4). For example, the decrease in the AUC for DeepRisk is more than two times slower than that for pruning and thresholding method for breast cancer (Fig. 4d). In summary, compared with the pruning and thresholding method, and the lasso method, the results showed that DeepRisk is more robust for identifying the variation in significant SNPs for disease risk prediction.

## 4 Discussion

In the past 25 years, the clinical potential of identifying individuals at high disease risk has attracted widespread attention. The success of PRS depends not only on genome-wide association study (GWAS) data but also on the development of risk models. More accurate disease risk prediction may lead to better customized screening, prevention and treatment. In this study, we propose a novel risk score calculation model and analyze the risk of four common diseases.

Alzheimer's disease, the most common form of dementia, accounts for approximately 60% of all cases of dementia [52]. At present, there are approximately 50 million AD patients worldwide, which has become one of the biggest public health challenges. Early diagnosis of AD may provide important personal and economic benefits [53]. DeepRisk_AD_ identified 7.90% of the population at greater than fivefold risk, and the top 1% of the population had more than 8.97-fold risk (Table S2). For AD, the proportion of individuals at high risk was much greater than that for the other three diseases, possibly because the number of patients in the AD dataset was too small. As a risk warning, this score can be a sign of early intervention for high-risk people. The results of a large, long-term, randomized controlled trial showed that multifaceted interventions, including diet, exercise, cognitive training, and vascular risk monitoring, can improve or maintain cognitive function in individuals at risk of developing general dementia (60-77 years old) [54].

As a global disease with accelerating incidence in newly industrialized countries, the burden of IBD remains high, and its prevalence surpasses 0.3% [55,56]. DeepRisk_IBD_ identified 4.19% of the population at greater than threefold risk, and the top 1% had more than 3.75-fold risk (Table S2). Although existing studies have shown that factors such as diet, probiotics, and antibiotics are related to the development of IBD, additional studies are needed to explore the mechanisms that can help to prevent IBD [55,57]. Identifying high-risk populations by DeepRisk may lead to new opportunities for large-scale population epidemiological studies to assess novel preventive therapies.

Type 2 diabetes is an expanding global health problem and places an enormous burden on health-care systems [58]. DeepRisk_T2D_ identified 4.10% of the population at greater than threefold risk, and the top 1% had more than 3.70-fold risk (Table S2). A study from Finland showed that increasing physical activity and an intensive lifestyle may substantially reduce the incidence of type 2 diabetes in high-risk individuals [59]. Therefore, the high-risk population determined by our DeepRisk_T2D_ can be prevented early to reduce the risk of type 2 diabetes.

Breast cancer is the second leading cause of death from cancer in women and affects one in twenty people globally and as many as one in eight people in high-income countries [60]. Fortunately, studies have shown that early detection and treatment can considerably improve patient outcomes [60,61]. DeepRisk_BC_ identified 1.59% of the population at greater than threefold risk, and the top 1% had more than 3.28-fold risk (Table S2). Although the use of current prevention methods continues to increase, with the increasing use of screening methods, the serious side effects of chemical and biological prevention still cannot be ignored [62]. An assessment of those with high deep polygenic risk scores may provide an opportunity to adopt these interventions more precisely.

These results showed that DeepRisk can identify individuals at increased risk for several common diseases. In addition to using the genotype information available at birth as a predictor, additional features can be added to the predictor over time. As such, the accuracy of DeepRisk may improve.

## 5 Conclusion

DeepRisk is a novel method for calculating risk scores for common diseases based on genome-wide genotype data. The risk of an individual can be assessed based on genotypic information long before the emergence of other risk factors. Based on the data of 488,377 individuals in the UK Biobank dataset, the results showed that DeepRisk consistently performed best compared to the state-of-the-art methods on all four Alzheimer's disease, inflammatory bowel disease, type 2 diabetes and breast cancer cases and achieves highest 8% improvement. We believe that DeepRisk will be able to provide more diverse and effective means for the prediction of public disease risk and play a positive role in the early prevention and customization of diagnosis and treatment processes for individuals.

## Availability of data and materials

The dataset supporting the conclusions of this article is available from the UK Biobank at https://www.ukbiobank.ac.uk/

The implemented code is available online at https://github.com/23AIBox/23AIBox-DeepRisk.

## Declaration of competing interest

The authors declare that they have no conflicts of interest in this work.

## Appendix Supplementary materials

Image, application 1
