# Supplementary material for: DeepRisk: A deep learning approach for genome-wide assessment of common disease risk
Source: Fundam Res. 2024 Mar 19;4(4):752–60. doi: 10.1016/j.fmre.2024.02.015 (PMC11330112; doi:10.1016/j.fmre.2024.02.015)
Supplement: Supplementary file 1 [file mmc1.docx]

Supporting Information

**DeepRisk: A Deep Learning Approach for Genome-wide Assessment of Common Disease Risk**

Jiajie Peng,^*^ Zhijie Bao, Jingyi Li, Ruijiang Han, Yuxian Wang, Lu Han, Jinghao Peng, Tao Wang, Jianye Hao, Zhongyu Wei^*^, Xuequn Shang^*^

**DeepRisk model with additional features**

On the basis of the DeepRisk model, SNP features are firstly transformed into gene features by the partial connected layer. The number of additional features is much smaller than the number of genes. The contribution of additional features in the neural network will be limited and may have little effect on the final prediction performance, if we concatenate the additional features with gene features directly. Therefore, the normalized covariate features are first expanded to the same dimension as the gene features by the fully connected layer. Then, the gene features and additional features are concatenated together, and the BiLSTM layer is used for feature extraction. Finally, we concatenate the output of the BiLSTM layer and the low-dimensional additional features before the fully connected layer to get the final deep polygenic risk score with additional features (**Figure. S6)**.

**Parameters of DeepRisk model**

Inspired by previous study, *r^2^* is considered as a tunable parameter, which is trained on the training set. LD window size is set as 250KB. The parameters of the BiLSTM are as follows. We set the hidden units of each unidirectional LSTM as 4. In order to avoid overfitting, we set the penalty coefficient as 0.001 for L2 regularization and dropout rate as 0.25 on BiLSTM layer. We run 20 epochs with 512 samples for each batch. We use AdamW algorithm and set an initial learning rate as 0.001 to optimize the weighted binary cross-entropy loss.

**Introduction of two compare methods**

**Pruning and thresholding**

The pruning and thresholding method calculates polygenic risk score based on the weights and SNP genotypes. Weights are generally assigned to each genetic variant according to the strength of their association with disease risk (effect estimate) from recent GWAS. The genotypes are scored based on how many risk alleles they have for each variant (for example, zero, one, or two). Given an individual having $n$ SNPs, the PRS can be obtained as follows:

$$PRS=\sum_{i=1}^{n} \beta_{i}x_{i} (1)$$

Where the $\beta_{i}$ is the effect estimate of SNP $i$, $x_{i}$is the genotype of SNP $i$. The PRS of the pruning and thresholding method is evaluated based on a logistic regression model with the disease as the outcome. The loss function is the binary cross-entropy loss with class weights.

**Lasso method**

The lasso method calculates PRS based on SNP genotypes. The PRS of the lasso method is obtained based on a LASSO logistic regression model. The loss function is the binary cross-entropy loss with class weights. We used AdamW algorithm and set an initial learning rate as 0.001 to optimize the weighted binary cross-entropy loss.

**
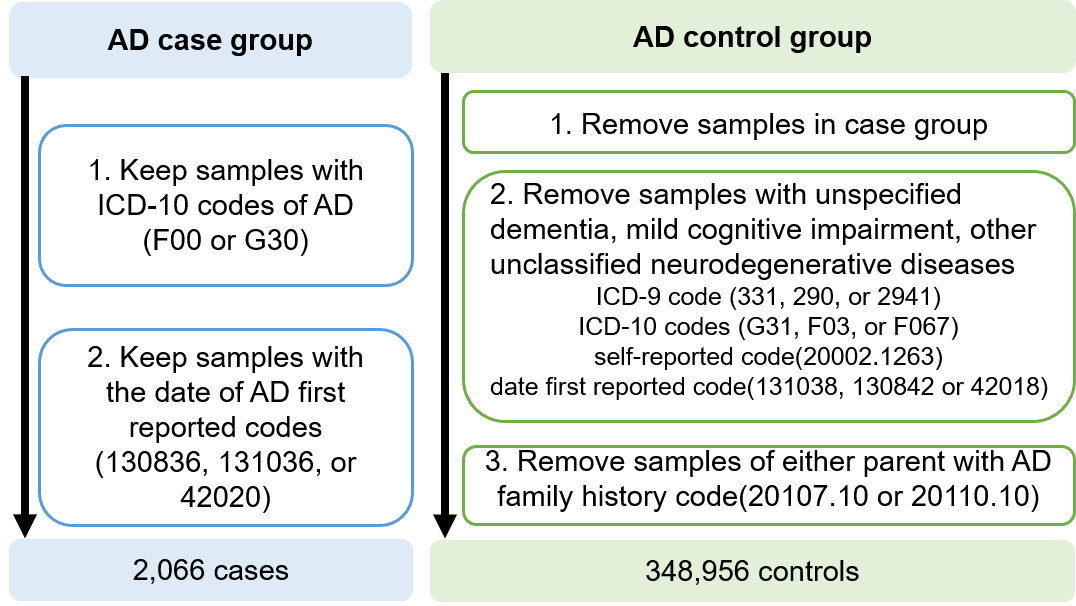
**

Figure. S1. Determination of case group and control group for Alzheimer's disease.


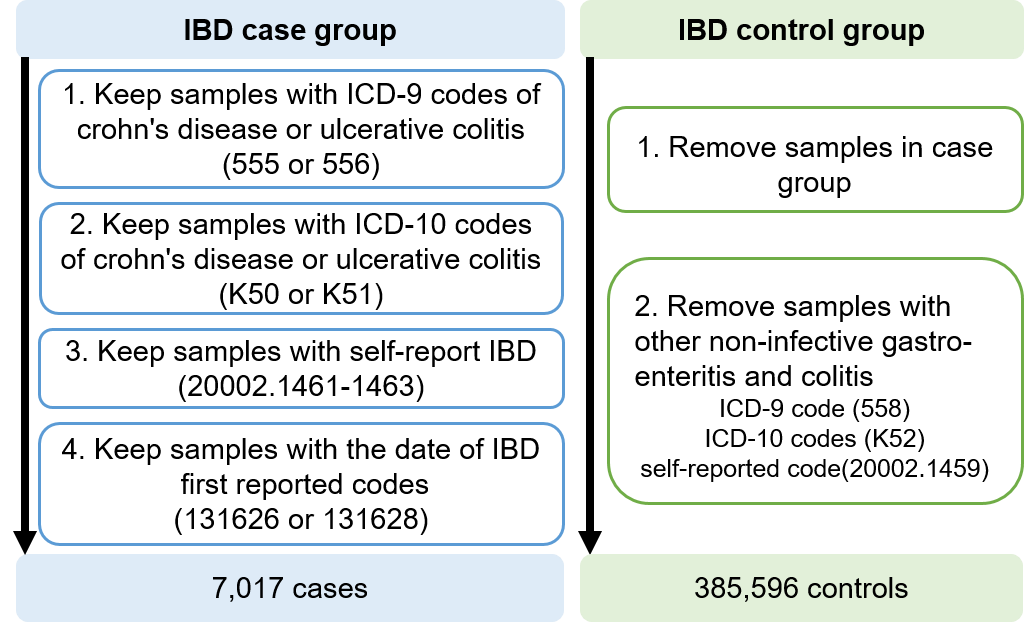


Figure. S2. Determination of case group and control group for inflammatory bowel disease.


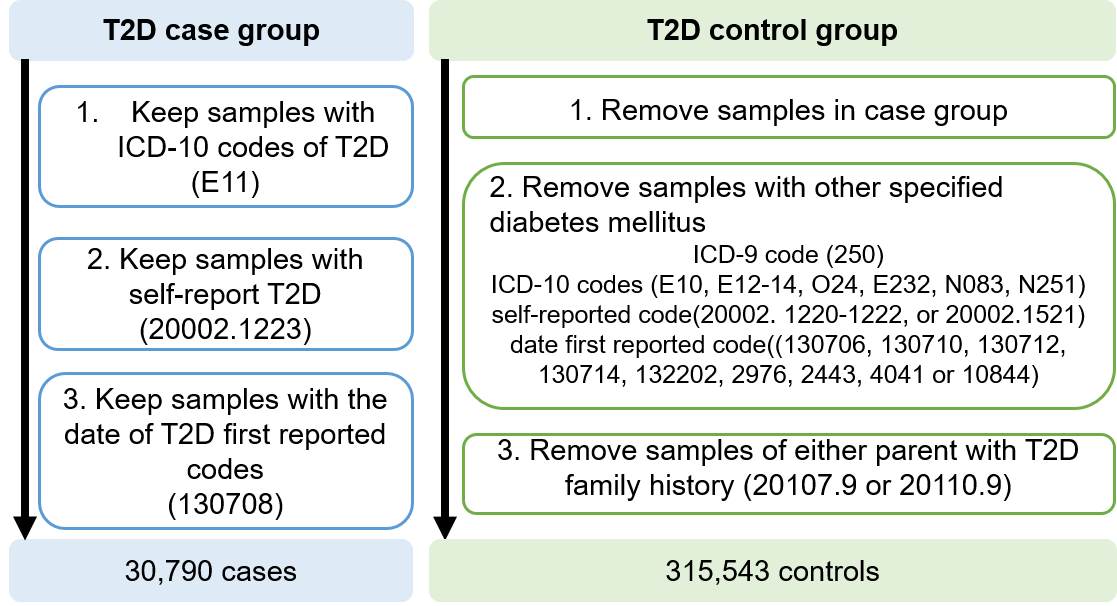


Figure. S3. Determination of case group and control group for type 2 diabetes.


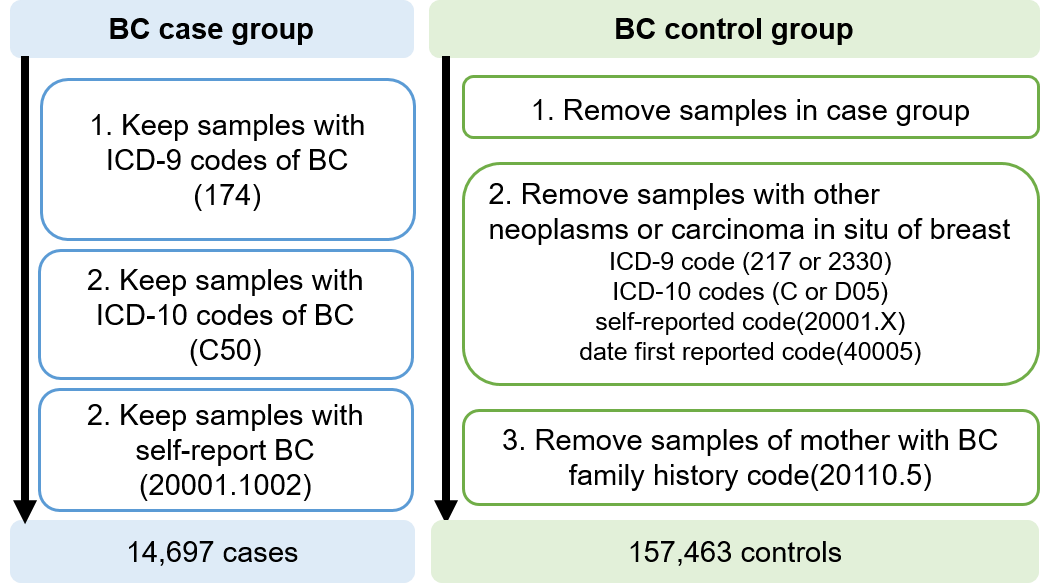


Figure. S4. Determination of case group and control group for breast cancer.


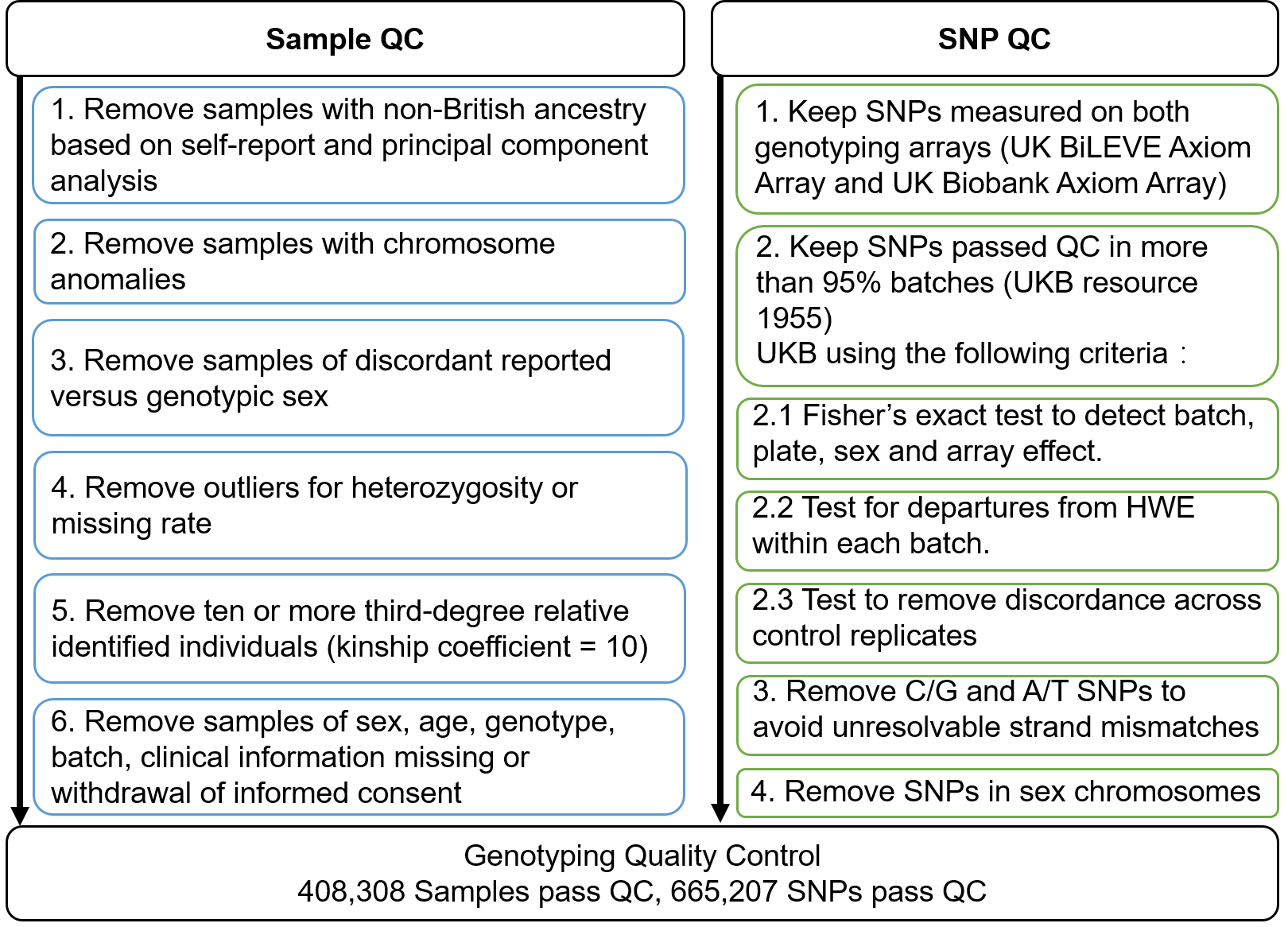


Figure. S5. Quality control process of the UK Biobank genotype dataset. The left side includes the sample QC steps. The right side includes the SNP QC steps.


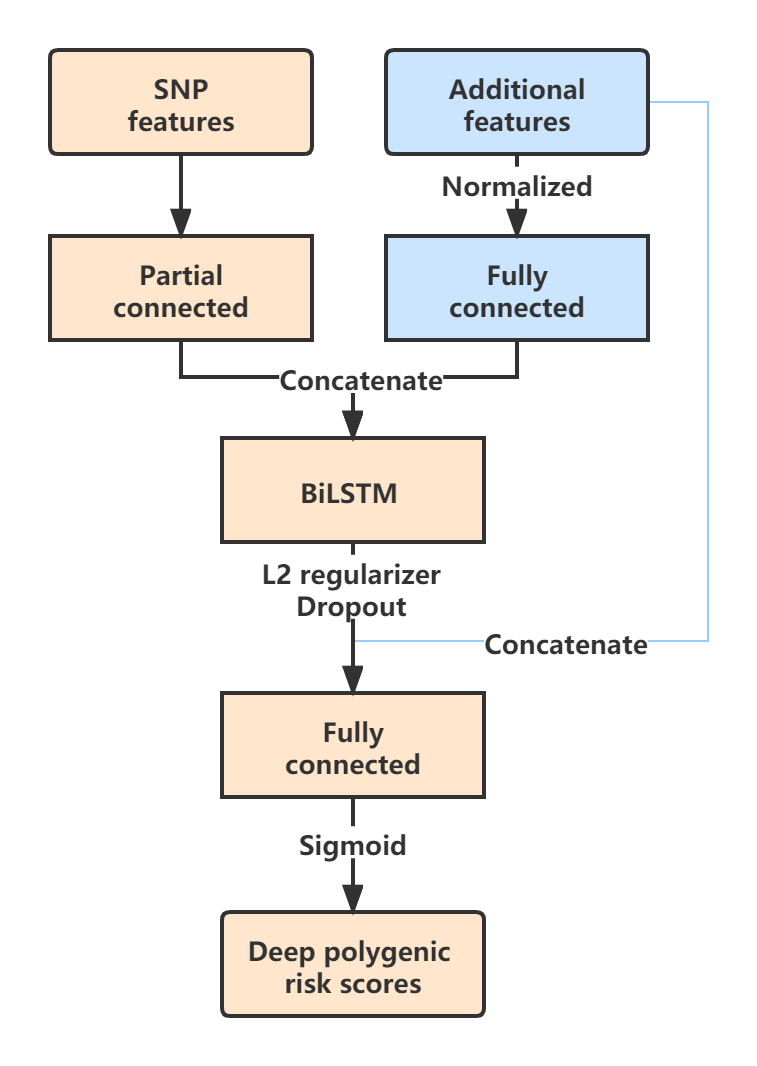


Figure. S6. The model architecture of DeepRisk with additional features.

The deep polygenic risk score with additional features is determined using the DeepRisk model with genotype, age, sex, genotype measurement batch, genotype array, region of assessment center, Townsend Deprivation index at recruitment, education-qualifications, the first four of genetic principal components. The blue components in this figure are used to incorporate the additional features with genotype information.


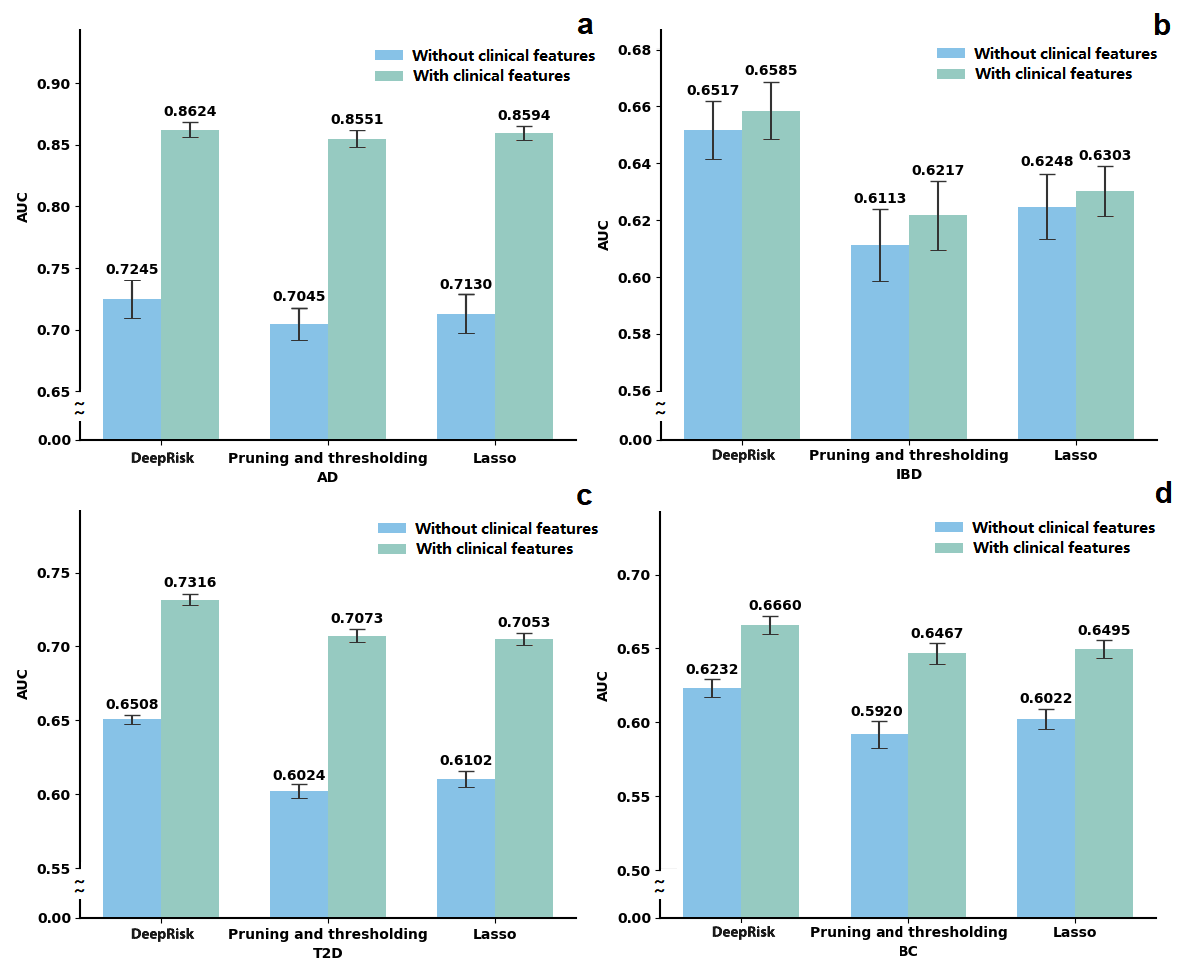


**Figure. S7. The results of risk prediction using genotype only and genotype with additional features for Alzheimer’s disease (a), inflammatory bowel disease (b), type 2 diabetes (c) and breast cancer (d). Compared with the pruning and thresholding method, and lasso model, the results show that the DeepRisk achieves the best performance on all the four diseases.**

**
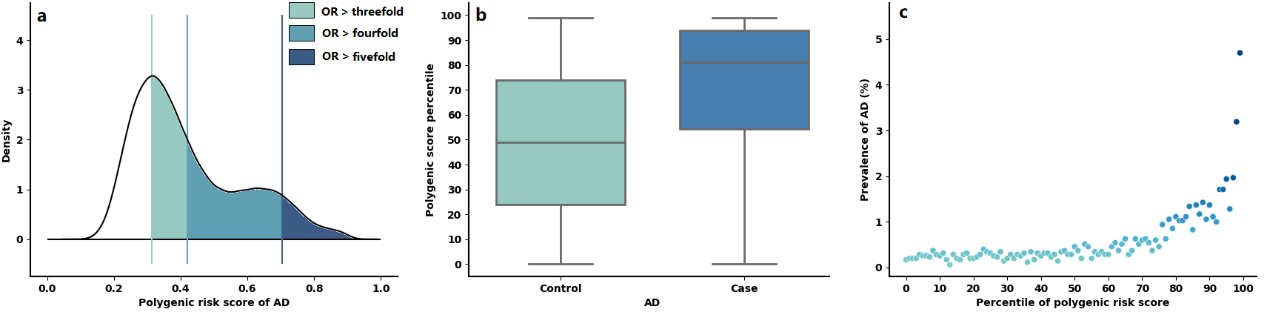
**

Figure. S8. Risk analysis for AD based on DeepRisk.

**(a)**, Distribution of deep polygenic risk score of AD in the UK Biobank dataset. The *X* axis represents deep polygenic risk score of AD. Shading reflects the proportion of the population with three-, four-, and fivefold increased risk versus the remainder of the population. **(b)**, Deep polygenic risk score percentile among AD cases versus controls in the UK Biobank dataset. In each boxplot, the horizontal lines reflect the median. The top and bottom of each box reflect the quartile range. The whiskers reflect the maximum and minimum values within each group. **(c)**, Prevalence of AD according to 100 groups of the dataset binned according to the percentile of the deep polygenic risk score of AD.


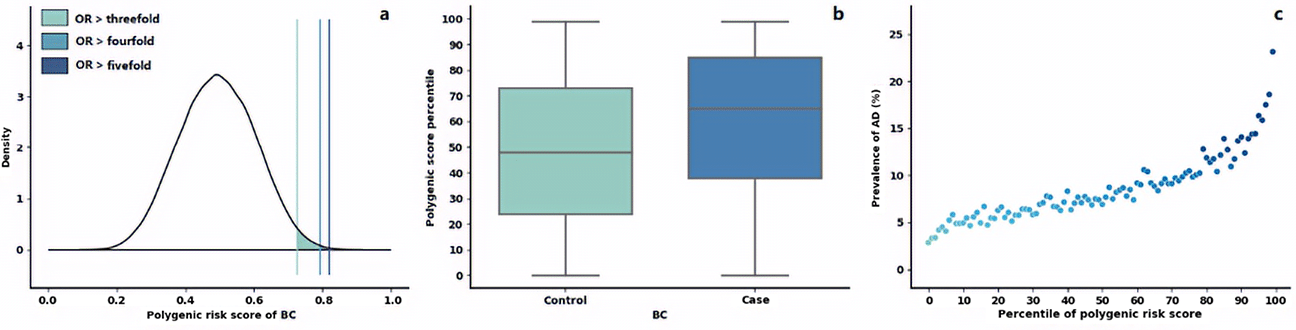


Figure. S9. Risk analysis for BC based on DeepRisk.

**(a)**, Distribution of deep polygenic risk score of BC in the UK Biobank dataset. The x axis represents deep polygenic risk score of BC. Shading reflects the proportion of the population with three-, four-, and fivefold increased risk versus the remainder of the population. **(b)**, Deep polygenic risk score percentile among BC cases versus controls in the UK Biobank dataset. In each boxplot, the horizontal lines reflect the median. The top and bottom of each box reflect the quartile range. The whiskers reflect the maximum and minimum values within each group. **(c)**, Prevalence of BC according to 100 groups of the dataset binned according to the percentile of the deep polygenic risk score of BC.


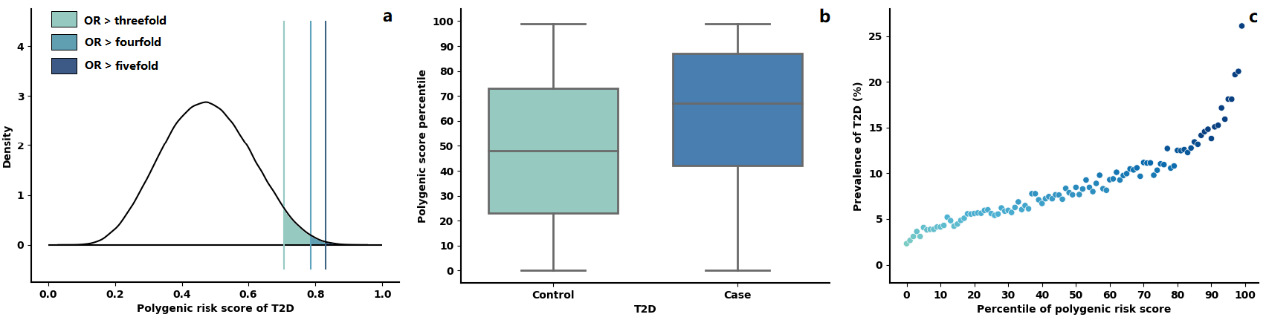


Figure. S10. Risk analysis for T2D based on DeepRisk.

**(a)**, Distribution of deep polygenic risk score of T2D in the UK Biobank dataset. The x axis represents deep polygenic risk score of T2D. Shading reflects the proportion of the population with three-, four-, and fivefold increased risk versus the remainder of the population. **(b)**, Deep polygenic risk score percentile among T2D cases versus controls in the UK Biobank dataset. In each boxplot, the horizontal lines reflect the median. The top and bottom of each box reflect the quartile range. The whiskers reflect the maximum and minimum values within each group. **(c)**, Prevalence of T2D according to 100 groups of the dataset binned according to the percentile of the deep polygenic risk score of T2D.

**Table. S. 1. An illustration example of calculating odds ratio.** Odds ratios are calculated by comparing those with high deep polygenic risk score to the remainder of the population. OR= ($\mathbf{T}_{\boldsymbol{D}}\mathbf{R}_{\boldsymbol{E}}$)/($\mathbf{T}_{\boldsymbol{E}}\mathbf{R}_{\boldsymbol{D}}$).

| Population/Phenotype | Diseased | Healthy |
| --- | --- | --- |
| Top 20% of distribution | $T_{D}$ | $T_{E}$ |
| Remaining 80% | $R_{D}$ | $R_{E}$ |

Table. S. 2. Odds ratio of a high deep polygenic risk score. The polygenic risk score is generated based on the genotype information in the UKB dataset. The calculation of odds ratio (OR) is based on the polygenetic risk score generated under the best performance parameters of each method.

| **High deep polygenic risk score definition** | **Reference group** | **OR based on DeepRisk** | **OR based on lasso model** |
| --- | --- | --- | --- |
| **Alzheimer's disease** |  |  |  |
| Top 20% of distribution | Remaining 80% | 4.64 | 2.15 |
| Top 10% of distribution | Remaining 90% | 4.70 | 2.45 |
| Top 5% of distribution | Remaining 95% | 5.55 | 2.81 |
| Top 1% of distribution | Remaining 99% | 8.97 | 3.58 |
| **Inflammatory bowel disease** |  |  |  |
| Top 20% of distribution | Remaining 80% | 2.39 | 1.58 |
| Top 10% of distribution | Remaining 90% | 2.56 | 1.78 |
| Top 5% of distribution | Remaining 95% | 2.87 | 1.93 |
| Top 1% of distribution | Remaining 99% | 3.75 | 2.15 |
| **Type 2 diabetes** |  |  |  |
| Top 20% of distribution | Remaining 80% | 2.41 | 1.72 |
| Top 10% of distribution | Remaining 90% | 2.60 | 1.78 |
| Top 5% of distribution | Remaining 95% | 2.92 | 1.84 |
| Top 1% of distribution | Remaining 99% | 3.70 | 1.81 |
| **Breast cancer** |  |  |  |
| Top 20% of distribution | Remaining 80% | 2.12 | 1.22 |
| Top 10% of distribution | Remaining 90% | 2.29 | 1.30 |
| Top 5% of distribution | Remaining 95% | 2.56 | 1.43 |
| Top 1% of distribution | Remaining 99% | 3.29 | 1.68 |

Table. S. 3. The comparison of risk prediction performance for Alzheimer's disease using only genotype information. Bold numbers indicate the best performance. ‘-’ Indicates that *r^2^* is not used for SNP selection.

| **Tuning Parameter** | **N Variants in Score** | **N Genes in Score** | **DeepRisk AUC** | **Pruning and thresholding AUC** | **Lasso AUC** |
| --- | --- | --- | --- | --- | --- |
| *P*<5×10^-3^; - | 3834 | 1454 | 0.7145 | 0.6354 | 0.7005 |
| *P*<5×10^-3^; *r^2^*<0.8 | 3291 | 1448 | 0.7170 | 0.6681 | 0.7037 |
| *P*<5×10^-3^; *r^2^*<0.6 | 3048 | 1444 | 0.7146 | 0.6585 | 0.7029 |
| *P*<5×10^-3^; *r^2^*<0.4 | 2811 | 1439 | 0.7144 | 0.6440 | 0.7002 |
| *P*<5×10^-3^; *r^2^*<0.2 | 2577 | 1432 | 0.6995 | 0.6023 | 0.6831 |
| *P*<5×10^-4^; - | 771 | 291 | **0.7245** | 0.6765 | 0.7108 |
| *P*<5×10^-4^; *r^2^*<0.8 | 606 | 287 | 0.7237 | 0.6983 | **0.7130** |
| *P*<5×10^-4^; *r^2^*<0.6 | 550 | 287 | 0.7212 | 0.6941 | 0.7105 |
| *P*<5×10^-4^; *r^2^*<0.4 | 491 | 287 | 0.7186 | 0.6882 | 0.7098 |
| *P*<5×10^-4^; *r^2^*<0.2 | 440 | 288 | 0.7035 | 0.6544 | 0.6929 |
| *P*<5×10^-5^; - | 259 | 57 | 0.7212 | 0.6972 | 0.7100 |
| *P*<5×10^-5^; *r^2^*<0.8 | 194 | 57 | 0.7205 | 0.7039 | 0.7095 |
| *P*<5×10^-5^; *r^2^*<0.6 | 169 | 57 | 0.7187 | 0.7016 | 0.7078 |
| *P*<5×10^-5^; *r^2^*<0.4 | 146 | 58 | 0.7169 | 0.6998 | 0.7086 |
| *P*<5×10^-5^; *r^2^*<0.2 | 115 | 57 | 0.7029 | 0.6747 | 0.6924 |
| *P*<5×10^-6^; *-* | 131 | 25 | 0.7180 | **0.7045** | 0.7093 |
| *P*<5×10^-6^; *r^2^*<0.8 | 105 | 25 | 0.7173 | 0.7034 | 0.7089 |
| *P*<5×10^-6^; *r^2^*<0.6 | 88 | 25 | 0.7154 | 0.7014 | 0.7074 |
| *P*<5×10^-6^; *r^2^*<0.4 | 77 | 25 | 0.7134 | 0.6996 | 0.7079 |
| *P*<5×10^-6^; *r^2^*<0.2 | 62 | 24 | 0.7010 | 0.6767 | 0.6924 |

**Table. S. 4. The comparison of risk prediction performance for Alzheimer's disease using genotype information and additional features.** Bold numbers indicate the best performance. ‘-’ Indicates that *r^2^* is not used for SNP selection.

| **Tuning Parameter** | **N Variants in Score** | **N Genes in Score** | **DeepRisk with additional features AUC** | **Pruning and thresholding** **with additional features AUC** | **Lasso** **with additional features AUC** |
| --- | --- | --- | --- | --- | --- |
| *P*<5×10^-3^; *-* | 3834 | 1454 | 0.8586 | 0.8322 | 0.8477 |
| *P*<5×10^-3^; *r^2^*<0.8 | 3291 | 1448 | 0.8581 | 0.8419 | 0.8514 |
| *P*<5×10^-3^; *r^2^*<0.6 | 3048 | 1444 | 0.8591 | 0.8387 | 0.8523 |
| *P*<5×10^-3^; *r^2^*<0.4 | 2811 | 1439 | 0.8588 | 0.8344 | 0.8540 |
| *P*<5×10^-3^; *r^2^*<0.2 | 2577 | 1432 | 0.8525 | 0.8237 | 0.8471 |
| *P*<5×10^-4^; *-* | 771 | 291 | **0.8624** | 0.8444 | 0.8591 |
| *P*<5×10^-4^; *r^2^*<0.8 | 606 | 287 | 0.8621 | 0.8519 | 0.8592 |
| *P*<5×10^-4^; *r^2^*<0.6 | 550 | 287 | 0.8615 | 0.8504 | 0.8592 |
| *P*<5×10^-4^; *r^2^*<0.4 | 491 | 287 | 0.8603 | 0.8484 | 0.8586 |
| *P*<5×10^-4^; *r^2^*<0.2 | 440 | 288 | 0.8538 | 0.8370 | 0.8508 |
| *P*<5×10^-5^; *-* | 259 | 57 | 0.8614 | 0.8517 | 0.8594 |
| *P*<5×10^-5^; *r^2^*<0.8 | 194 | 57 | 0.8615 | 0.8546 | 0.8592 |
| *P*<5×10^-5^; *r^2^*<0.6 | 169 | 57 | 0.8612 | 0.8537 | 0.8588 |
| *P*<5×10^-5^; *r^2^*<0.4 | 146 | 58 | 0.8605 | 0.8529 | 0.8581 |
| *P*<5×10^-5^; *r^2^*<0.2 | 115 | 57 | 0.8537 | 0.8439 | 0.8508 |
| *P*<5×10^-6^; *-* | 131 | 25 | 0.8608 | **0.8551** | 0.8593 |
| *P*<5×10^-6^; *r^2^*<0.8 | 105 | 25 | 0.8610 | 0.8548 | **0.8594** |
| *P*<5×10^-6^; *r^2^*<0.6 | 88 | 25 | 0.8605 | 0.8540 | 0.8587 |
| *P*<5×10^-6^; *r^2^*<0.4 | 77 | 25 | 0.8595 | 0.8531 | 0.8584 |
| *P*<5×10^-6^; *r^2^*<0.2 | 62 | 24 | 0.8536 | 0.8448 | 0.8509 |

**Table. S. 5.** **The comparison of risk prediction performance for inflammatory bowel disease using only genotype information.** Bold numbers indicate the best performance. ‘-’ Indicates that *r^2^* is not used for SNP selection.

| **Tuning Parameter** | **N Variants in Score** | **N Genes in Score** | **DeepRisk AUC** | **Pruning and thresholding AUC** | **Lasso AUC** |
| --- | --- | --- | --- | --- | --- |
| *P*<5×10^-3^; *-* | 7848 | 2082 | 0.6506 | 0.5817 | 0.6157 |
| *P*<5×10^-3^; *r^2^*<0.8 | 6188 | 2070 | 0.6503 | 0.6005 | 0.6235 |
| *P*<5×10^-3^; *r^2^*<0.6 | 5626 | 2065 | 0.6501 | 0.6040 | 0.6244 |
| *P*<5×10^-3^; *r^2^*<0.4 | 5041 | 2055 | 0.6478 | 0.6031 | 0.6211 |
| *P*<5×10^-3^; *r^2^*<0.2 | 4423 | 2035 | 0.6417 | 0.5962 | 0.6172 |
| *P*<5×10^-4^; *-* | 2481 | 542 | **0.6517** | 0.5783 | 0.6166 |
| *P*<5×10^-4^; *r^2^*<0.8 | 1689 | 533 | 0.6493 | 0.5986 | 0.6242 |
| *P*<5×10^-4^; *r^2^*<0.6 | 1438 | 537 | 0.6490 | 0.6062 | **0.6248** |
| *P*<5×10^-4^; *r^2^*<0.4 | 1219 | 535 | 0.6477 | **0.6113** | 0.6241 |
| *P*<5×10^-4^; *r^2^*<0.2 | 1018 | 525 | 0.6420 | 0.6099 | 0.6200 |
| *P*<5×10^-5^; *-* | 1298 | 200 | 0.6477 | 0.5750 | 0.6192 |
| *P*<5×10^-5^; *r^2^*<0.8 | 813 | 198 | 0.6469 | 0.5919 | 0.6201 |
| *P*<5×10^-5^; *r^2^*<0.6 | 649 | 198 | 0.6477 | 0.6009 | 0.6226 |
| *P*<5×10^-5^; *r^2^*<0.4 | 535 | 197 | 0.6451 | 0.6087 | 0.6217 |
| *P*<5×10^-5^; *r^2^*<0.2 | 407 | 193 | 0.6410 | 0.6101 | 0.6184 |
| *P*<5×10^-6^; *-* | 892 | 112 | 0.6410 | 0.5728 | 0.6109 |
| *P*<5×10^-6^; *r^2^*<0.8 | 535 | 113 | 0.6406 | 0.5874 | 0.6176 |
| *P*<5×10^-6^; *r^2^*<0.6 | 418 | 113 | 0.6404 | 0.5957 | 0.6185 |
| *P*<5×10^-6^; *r^2^*<0.4 | 328 | 115 | 0.6380 | 0.6042 | 0.6153 |
| *P*<5×10^-6^; *r^2^*<0.2 | 250 | 113 | 0.6344 | 0.6062 | 0.6136 |

**Table. S. 6. The comparison of risk prediction performance for inflammatory bowel disease using genotype information and additional features.** Bold numbers indicate the best performance. ‘-’ Indicates that *r^2^* is not used for SNP selection.

| **Tuning Parameter** | **N Variants in Score** | **N Genes in Score** | **DeepRisk with additional features AUC** | **Pruning and thresholding** **with additional features AUC** | **Lasso** **with additional features AUC** |
| --- | --- | --- | --- | --- | --- |
| *P*<5×10^-3^; *-* | 7848 | 2082 | 0.6564 | 0.5959 | 0.5772 |
| *P*<5×10^-3^; *r^2^*<0.8 | 6188 | 2070 | 0.6567 | 0.6125 | 0.6186 |
| *P*<5×10^-3^; *r^2^*<0.6 | 5626 | 2065 | 0.6568 | 0.6156 | 0.5953 |
| *P*<5×10^-3^; *r^2^*<0.4 | 5041 | 2055 | 0.6551 | 0.6146 | 0.6114 |
| *P*<5×10^-3^; *r^2^*<0.2 | 4423 | 2035 | 0.6491 | 0.6083 | 0.6236 |
| *P*<5×10^-4^; *-* | 2481 | 542 | **0.6585** | 0.5929 | 0.6148 |
| *P*<5×10^-4^; *r^2^*<0.8 | 1689 | 533 | 0.6564 | 0.6104 | 0.6269 |
| *P*<5×10^-4^; *r^2^*<0.6 | 1438 | 537 | 0.6553 | 0.6172 | 0.6299 |
| *P*<5×10^-4^; *r^2^*<0.4 | 1219 | 535 | 0.6544 | **0.6217** | 0.6253 |
| *P*<5×10^-4^; *r^2^*<0.2 | 1018 | 525 | 0.6495 | 0.6206 | 0.6160 |
| *P*<5×10^-5^; *-* | 1298 | 200 | 0.6547 | 0.5897 | 0.6153 |
| *P*<5×10^-5^; *r^2^*<0.8 | 813 | 198 | 0.6549 | 0.6042 | 0.6211 |
| *P*<5×10^-5^; *r^2^*<0.6 | 649 | 198 | 0.6552 | 0.6120 | **0.6303** |
| *P*<5×10^-5^; *r^2^*<0.4 | 535 | 197 | 0.6521 | 0.6191 | 0.6293 |
| *P*<5×10^-5^; *r^2^*<0.2 | 407 | 193 | 0.6492 | 0.6206 | 0.6283 |
| *P*<5×10^-6^; *-* | 892 | 112 | 0.6485 | 0.5880 | 0.6227 |
| *P*<5×10^-6^; *r^2^*<0.8 | 535 | 113 | 0.6483 | 0.6004 | 0.6174 |
| *P*<5×10^-6^; *r^2^*<0.6 | 418 | 113 | 0.6483 | 0.6074 | 0.6228 |
| *P*<5×10^-6^; *r^2^*<0.4 | 328 | 115 | 0.6459 | 0.6149 | 0.6267 |
| *P*<5×10^-6^; *r^2^*<0.2 | 250 | 113 | 0.6427 | 0.6167 | 0.6238 |

**Table. S. 7. The comparison of risk prediction performance for type 2 diabetes using only genotype information.** Bold numbers indicate the best performance**.** ‘-’ Indicates that *r^2^* is not used for SNP selection.

| **Tuning Parameter** | **N Variants in Score** | **N Genes in Score** | **DeepRisk AUC** | **Pruning and thresholding AUC** | **Lasso AUC** |
| --- | --- | --- | --- | --- | --- |
| *P*<5×10^-3^; *-* | 5968 | 1890 | **0.6508** | 0.5899 | **0.6102** |
| *P*<5×10^-3^; *r^2^*<0.8 | 5069 | 1878 | 0.6501 | **0.6024** | 0.6087 |
| *P*<5×10^-3^; *r^2^*<0.6 | 4588 | 1865 | 0.6488 | 0.5979 | 0.6080 |
| *P*<5×10^-3^; *r^2^*<0.4 | 4203 | 1855 | 0.6476 | 0.5958 | 0.6071 |
| *P*<5×10^-3^; *r^2^*<0.2 | 3769 | 1837 | 0.6440 | 0.5863 | 0.6032 |
| *P*<5×10^-4^; *-* | 1384 | 481 | 0.6346 | 0.5885 | 0.6039 |
| *P*<5×10^-4^; *r^2^*<0.8 | 1059 | 478 | 0.6338 | 0.6023 | 0.6029 |
| *P*<5×10^-4^; *r^2^*<0.6 | 919 | 477 | 0.6335 | 0.6006 | 0.6033 |
| *P*<5×10^-4^; *r^2^*<0.4 | 816 | 475 | 0.6321 | 0.5981 | 0.6012 |
| *P*<5×10^-4^; *r^2^*<0.2 | 700 | 472 | 0.6300 | 0.5921 | 0.5993 |
| *P*<5×10^-5^; *-* | 538 | 144 | 0.6244 | 0.5950 | 0.5991 |
| *P*<5×10^-5^; *r^2^*<0.8 | 380 | 144 | 0.6243 | 0.6013 | 0.5984 |
| *P*<5×10^-5^; *r^2^*<0.6 | 309 | 143 | 0.6232 | 0.6017 | 0.5992 |
| *P*<5×10^-5^; *r^2^*<0.4 | 263 | 143 | 0.6226 | 0.6016 | 0.5980 |
| *P*<5×10^-5^; *r^2^*<0.2 | 219 | 142 | 0.6217 | 0.6007 | 0.5971 |
| *P*<5×10^-6^; *-* | 291 | 70 | 0.6157 | 0.5930 | 0.5946 |
| *P*<5×10^-6^; *r^2^*<0.8 | 191 | 70 | 0.6153 | 0.5948 | 0.5946 |
| *P*<5×10^-6^; *r^2^*<0.6 | 148 | 70 | 0.6146 | 0.5954 | 0.5948 |
| *P*<5×10^-6^; *r^2^*<0.4 | 123 | 70 | 0.6135 | 0.5962 | 0.5939 |
| *P*<5×10^-6^; *r^2^*<0.2 | 102 | 70 | 0.6138 | 0.5988 | 0.5942 |

**Table. S. 8. The comparison of risk prediction performance for type 2 diabetes using genotype information and additional features.** Bold numbers indicate the best performance. ‘-’ Indicates that *r^2^* is not used for SNP selection.

| **Tuning Parameter** | **N Variants in Score** | **N Genes in Score** | **DeepRisk with additional features AUC** | **Pruning and thresholding** **with additional features AUC** | **Lasso** **with additional features AUC** |
| --- | --- | --- | --- | --- | --- |
| *P*<5×10^-3^; *-* | 5968 | 1890 | **0.7316** | 0.7010 | 0.6953 |
| *P*<5×10^-3^; *r^2^*<0.8 | 5069 | 1878 | 0.7306 | 0.7065 | 0.6991 |
| *P*<5×10^-3^; *r^2^*<0.6 | 4588 | 1865 | 0.7302 | 0.7042 | 0.7018 |
| *P*<5×10^-3^; *r^2^*<0.4 | 4203 | 1855 | 0.7291 | 0.7031 | 0.6990 |
| *P*<5×10^-3^; *r^2^*<0.2 | 3769 | 1837 | 0.7275 | 0.6993 | 0.6944 |
| *P*<5×10^-4^; *-* | 1384 | 481 | 0.7226 | 0.7011 | **0.7053** |
| *P*<5×10^-4^; *r^2^*<0.8 | 1059 | 478 | 0.7224 | **0.7073** | 0.7051 |
| *P*<5×10^-4^; *r^2^*<0.6 | 919 | 477 | 0.7225 | 0.7063 | 0.7047 |
| *P*<5×10^-4^; *r^2^*<0.4 | 816 | 475 | 0.7218 | 0.7049 | 0.7036 |
| *P*<5×10^-4^; *r^2^*<0.2 | 700 | 472 | 0.7206 | 0.7020 | 0.7033 |
| *P*<5×10^-5^; *-* | 538 | 144 | 0.7178 | 0.7044 | 0.7033 |
| *P*<5×10^-5^; *r^2^*<0.8 | 380 | 144 | 0.7174 | 0.7071 | 0.7045 |
| *P*<5×10^-5^; *r^2^*<0.6 | 309 | 143 | 0.7173 | 0.7070 | 0.7038 |
| *P*<5×10^-5^; *r^2^*<0.4 | 263 | 143 | 0.7170 | 0.7066 | 0.7041 |
| *P*<5×10^-5^; *r^2^*<0.2 | 219 | 142 | 0.7164 | 0.7059 | 0.7031 |
| *P*<5×10^-6^; *-* | 291 | 70 | 0.7135 | 0.7036 | 0.7029 |
| *P*<5×10^-6^; *r^2^*<0.8 | 191 | 70 | 0.7132 | 0.7043 | 0.7033 |
| *P*<5×10^-6^; *r^2^*<0.6 | 148 | 70 | 0.7129 | 0.7044 | 0.7028 |
| *P*<5×10^-6^; *r^2^*<0.4 | 123 | 70 | 0.7124 | 0.7045 | 0.7023 |
| *P*<5×10^-6^; *r^2^*<0.2 | 102 | 70 | 0.7125 | 0.7054 | 0.7024 |

**Table. S. 9. The comparison of risk prediction performance for breast cancer using only genotype information.** Bold numbers indicate the best performance. ‘-’ Indicates that *r^2^* is not used for SNP selection.

| **Tuning Parameter** | **N Variants in Score** | **N Genes in Score** | **DeepRisk AUC** | **Pruning and thresholding AUC** | **Lasso AUC** |
| --- | --- | --- | --- | --- | --- |
| *P*<5×10^-3^; *-* | 3830 | 1553 | **0.6232** | 0.5602 | **0.6022** |
| *P*<5×10^-3^; *r^2^*<0.8 | 3392 | 1546 | 0.6216 | 0.5551 | 0.6010 |
| *P*<5×10^-3^; *r^2^*<0.6 | 3156 | 1544 | 0.6217 | 0.5494 | 0.6007 |
| *P*<5×10^-3^; *r^2^*<0.4 | 2968 | 1544 | 0.6199 | 0.5437 | 0.5977 |
| *P*<5×10^-3^; *r^2^*<0.2 | 2736 | 1537 | 0.6190 | 0.5353 | 0.5965 |
| *P*<5×10^-4^; *-* | 662 | 285 | 0.6161 | 0.5720 | 0.5980 |
| *P*<5×10^-4^; *r^2^*<0.8 | 527 | 285 | 0.6151 | 0.5840 | 0.5982 |
| *P*<5×10^-4^; *r^2^*<0.6 | 473 | 285 | 0.6147 | 0.5799 | 0.5976 |
| *P*<5×10^-4^; *r^2^*<0.4 | 435 | 284 | 0.6138 | 0.5743 | 0.5952 |
| *P*<5×10^-4^; *r^2^*<0.2 | 383 | 284 | 0.6139 | 0.5653 | 0.5944 |
| *P*<5×10^-5^; *-* | 182 | 65 | 0.6078 | **0.5920** | 0.5940 |
| *P*<5×10^-5^; *r^2^*<0.8 | 153 | 65 | 0.6072 | 0.5903 | 0.5931 |
| *P*<5×10^-5^; *r^2^*<0.6 | 134 | 64 | 0.6070 | 0.5882 | 0.5926 |
| *P*<5×10^-5^; *r^2^*<0.4 | 116 | 64 | 0.6061 | 0.5856 | 0.5909 |
| *P*<5×10^-5^; *r^2^*<0.2 | 91 | 64 | 0.6055 | 0.5820 | 0.5888 |
| *P*<5×10^-6^; *-* | 91 | 28 | 0.6004 | 0.5889 | 0.5889 |
| *P*<5×10^-6^; *r^2^*<0.8 | 72 | 28 | 0.5997 | 0.5878 | 0.5875 |
| *P*<5×10^-6^; *r^2^*<0.6 | 61 | 27 | 0.5996 | 0.5869 | 0.5873 |
| *P*<5×10^-6^; *r^2^*<0.4 | 47 | 27 | 0.5988 | 0.5874 | 0.5847 |
| *P*<5×10^-6^; *r^2^*<0.2 | 36 | 27 | 0.5983 | 0.5869 | 0.5839 |

**Table. S. 10. The comparison of risk prediction performance for breast cancer using genotype information and additional features.** Bold numbers indicate the best performance. ‘-’ Indicates that *r^2^* is not used for SNP selection.

| Tuning Parameter | N Variants in Score | N Genes in Score | DeepRisk with additional features AUC | Pruning and thresholding with additional features AUC | Lasso with additional features AUC |
| --- | --- | --- | --- | --- | --- |
| *P*<5×10^-3^; *-* | 3830 | 1553 | **0.6660** | 0.6286 | 0.6478 |
| *P*<5×10^-3^; *r^2^*<0.8 | 3392 | 1546 | 0.6654 | 0.6262 | 0.6352 |
| *P*<5×10^-3^; *r^2^*<0.6 | 3156 | 1544 | 0.6656 | 0.6238 | 0.6406 |
| *P*<5×10^-3^; *r^2^*<0.4 | 2968 | 1544 | 0.6642 | 0.6217 | 0.6430 |
| *P*<5×10^-3^; *r^2^*<0.2 | 2736 | 1537 | 0.6636 | 0.6189 | 0.6444 |
| *P*<5×10^-4^; *-* | 662 | 285 | 0.6622 | 0.6350 | 0.6485 |
| *P*<5×10^-4^; *r^2^*<0.8 | 527 | 285 | 0.6612 | 0.6422 | **0.6495** |
| *P*<5×10^-4^; *r^2^*<0.6 | 473 | 285 | 0.6609 | 0.6398 | 0.6482 |
| *P*<5×10^-4^; *r^2^*<0.4 | 435 | 284 | 0.6604 | 0.6366 | 0.6472 |
| *P*<5×10^-4^; *r^2^*<0.2 | 383 | 284 | 0.6604 | 0.6318 | 0.6475 |
| *P*<5×10^-5^; *-* | 182 | 65 | 0.6566 | **0.6467** | 0.6480 |
| *P*<5×10^-5^; *r^2^*<0.8 | 153 | 65 | 0.6558 | 0.6454 | 0.6466 |
| *P*<5×10^-5^; *r^2^*<0.6 | 134 | 64 | 0.6561 | 0.6442 | 0.6467 |
| *P*<5×10^-5^; *r^2^*<0.4 | 116 | 64 | 0.6554 | 0.6425 | 0.6455 |
| *P*<5×10^-5^; *r^2^*<0.2 | 91 | 64 | 0.6551 | 0.6401 | 0.6439 |
| *P*<5×10^-6^; *-* | 91 | 28 | 0.6517 | 0.6452 | 0.6448 |
| *P*<5×10^-6^; *r^2^*<0.8 | 72 | 28 | 0.6513 | 0.6445 | 0.6435 |
| *P*<5×10^-6^; *r^2^*<0.6 | 61 | 27 | 0.6515 | 0.6441 | 0.6433 |
| *P*<5×10^-6^; *r^2^*<0.4 | 47 | 27 | 0.6507 | 0.6443 | 0.6419 |
| *P*<5×10^-6^; *r^2^*<0.2 | 36 | 27 | 0.6505 | 0.6440 | 0.6414 |

**Table. S. 11. Cases with small number of risk SNPs but high deep polygenic risk scores.** We define the SNPs with *P* value less than 5e-8 and the effect estimate (*β*) greater than 0 in GWAS as significant risk SNPs. ‘-’ Indicates that *r^2^* is not used for SNP selection.

| **Sample ID** | **Risk SNPs included in GWAS (n)** | **Mutations in Risk SNPs (n)** | **Pruning and thresholding method percentile score** | **DeepRisk percentile score** | **Odds ratio based on DeepRisk** |
| --- | --- | --- | --- | --- | --- |
| **Inflammatory bowel disease** |  |  |  |  |  |
| Individual 1 | 236 | 36 | 5 | 99 | 4.23 |
| Individual 2 | 236 | 42 | 5 | 99 | 4.64 |
| Individual 3 | 236 | 46 | 6 | 99 | 5.21 |
